# Crystal structure of di­acet­y­lene unveiled by X-ray and neutron diffraction, Raman spectroscopy and periodic DFT

**DOI:** 10.1107/S2052252525010486

**Published:** 2026-01-01

**Authors:** Larissa Lopes Cavalcante, Helen E. Maynard-Casely, Morgan L. Cable, Samuel G. Duyker, Edith C. Fayolle, Robert Hodyss, Brendan J. Kennedy, Tuan H. Vu, Courtney Ennis

**Affiliations:** a Department of Chemistry, University of Otago, Dunedin 9054, New Zealand; b Australian Centre for Neutron Scattering, ANSTO, Kirrawee, NSW 2232, Australia; c Jet Propulsion Laboratory, California Institute of Technology,Pasadena CA 91109 USA; d Sydney Analytical, Core Research Facilities, University of Sydney, NSW 2006, Australia; e School of Chemistry, University of Sydney, Sydney, NSW 2006, Australia; f MacDiarmid Institute for Advanced Materials and Nanotechnology, Wellington 6140 New Zealand; ESRF, France

**Keywords:** density functional theory, molecular crystals, intermolecular interactions, properties of solids, crystallization under non-ambient conditions, materials science, Dragonfly mission

## Abstract

Combining powder X-ray and neutron diffraction, the first crystallographic characterization of di­acet­y­lene (IUPAC designation 1,3-butadiyne) is presented and supplemented by Raman spectroscopy and periodic-DFT calculations. The structure is described in the space group *Pnma*, with *a* = 9.348 (2), *b* = 5.9890 (6), *c* = 5.6746 (11) Å and *V* = 317.69 (6) Å^3^ at 5 K, with four molecules in the unit cell resulting in a density of 1.0466 g cm^−3^. The di­acet­y­lene molecules are arranged in a layered structure dominated by C—H⋯π interactions, which leads to anisotropic thermal expansion behaviour. No structural phase transitions were observed across the 5–220 K tem­per­a­ture range at ambient pressure. The structural similarity with acetylene identifies it as a potential cocrystal component of particular importance for Titan’s surface chemistry in view of the upcoming NASA Dragonfly mission.

## Abstract 2

The crystal structure and thermal expansion behaviour of di­acet­y­lene were determined *via* diffraction methods and supplemented by spectroscopic techniques. The similarities to the structure of acetylene and implications for Titan *in situ* exploration are discussed.

## 1. Introduction

The crystallographic study of simple hydrocarbon species is of great importance for understanding molecular solids, particularly of those relevant to planetary systems (Maynard-Casely *et al.*, 2018 ▸; Maynard-Casely *et al.*, 2020 ▸; Vu *et al.*, 2022 ▸; Brand *et al.*, 2020 ▸). Investigating the phase behaviour, density and structural properties of these molecules is key to elucidating the physical processes and geochemical features observed in various bodies across the Solar System.

Saturn’s moon Titan has drawn significant attention in recent years due to its dense atmosphere, whose composition is a consequence of photochemical reactions initiated from its primary components: nitrogen and methane (Coustenis *et al.*, 2007 ▸; Hörst, 2017 ▸; Nixon, 2024 ▸). These reactions result in complex chemistry with over 20 molecular species identified in the upper atmosphere (Nixon, 2024 ▸). At lower altitudes, the reduced tem­per­a­tures facilitate the condensation and eventual precipitation of heavier hydrocarbons and nitrile molecules, such as acetylene (C_2_H_2_), hydrogen cyanide (HCN), benzene (C_6_H_6_) and 1,3-butadiyne (C_4_H_2_, hereafter referred to as di­acet­y­lene) onto Titan’s surface, where the tem­per­a­ture varies in the range 90–94 K (Barth, 2017 ▸).

Surface measurements from the Cassini–Huygens mission support the deposition of these small condensates, with images revealing a complex landscape of lakes and dunes where sediments accumulate over time (Lopes *et al.*, 2019 ▸). The fate of these compounds is of particular interest to NASA’s New Frontiers mission Dragonfly, scheduled to arrive in the 2030s, to investigate the surface composition of Titan (Maynard-Casely *et al.*, 2018 ▸; Barnes *et al.*, 2021 ▸; Cable *et al.*, 2021 ▸; Yu *et al.*, 2023 ▸; Yu *et al.*, 2024 ▸).

Among these condensates, di­acet­y­lene is potentially sig­nifi­cant in contributing to Titan’s atmospheric processes, where its abundance is estimated to be 15 ppb at 150 km altitude (Jolly *et al.*, 2010 ▸). It is thought to be formed from the photodissociation of acetylene (Smith *et al.*, 1998 ▸): 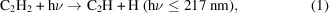

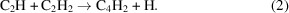
 Once formed, di­acet­y­lene acts as a precursor for higher polyynes by absorbing UV photons at relatively long wavelengths where the solar flux is significant (Barth, 2017 ▸). This absorption leads to the potential formation of radical species that are key to forming polycyclic aro­matic hydrocarbons (PAHs) (Huang *et al.*, 2010 ▸), and larger organic species that constitute the haze layers observed in Titan’s atmosphere (*e.g.* Fleury *et al.*, 2024 ▸).

At lower stratospheric altitudes, the condensation of di­acet­y­lene into icy grains is expected to occur around 80 km, where the tem­per­a­ture reaches 114 K (Barth, 2017 ▸; Yu *et al.*, 2023 ▸). Once formed, the ice descends and reaches the surface, where it is expected to remain solid (Yu *et al.*, 2023 ▸; Yu *et al.*, 2024 ▸). Therefore, understanding di­acet­y­lene’s solid phase behaviour is crucial for future *in situ* exploration of its interaction with other molecular species.

Early studies using polarized IR spectroscopy suggested an orthorhombic structure (space group *P*22_1_2_1_) at liquid N_2_ tem­per­a­ture (Freund & Halford, 1965 ▸; Khanna *et al.*, 1988 ▸), although this structure has remained unconfirmed by diffraction analysis. Here, we report the first characterization of the crystal structure of di­acet­y­lene by powder X-ray and neutron diffraction, along with its thermal expansion behaviour determined at ambient pressure and tem­per­a­tures ranging from 5 to 220 K. These results are further supported by comparison of ex­per­i­mental Raman spectroscopy with periodic density functional theory (p-DFT) calculated lattice vibrations from the determined crystal structure.

## 2. Methods

Diacetylene was synthesized according to the procedure described in the literature *via* the dehydrochlorination of 1,4-dichloro-2-butyne (ClCH_2_CCCH_2_Cl) (Zhou *et al.*, 2009 ▸). Here, 7.5 ml of ClCH_2_CCCH_2_Cl (AK Scientific, 99%) were added to 15 ml ethanol (C_2_H_5_OH; Lab Supply, 99.5%) and warmed to 353 K while stirring. Next, 50% aqueous sodium hydroxide (NaOH; Lab Supply, 97%) was added dropwise. A gas evolved and was bubbled through a 13% aqueous sodium hydroxide solution, then dried over calcium chloride (CaCl_2_; Lab Supply, 90%) and condensed in a liquid nitrogen trap (77 K).

### 2.1. Diffraction measurements and structure solution procedure

Neutron powder diffraction ex­per­i­ments were conducted on the Wombat high-intensity diffractometer at the Australian Centre for Neutron Scattering (Maynard-Casely *et al.*, 2025 ▸), utilizing a neutron wavelength of 2.41 Å. Confirmation of the wavelength was determined from a refinement of a NIST LaB_6_ standard, along with establishing a 2θ zero error. Diacetylene was condensed into a 6 mm diameter vanadium can affixed to a positioning system within a top-loading cryofurnace (Brochier, 1977 ▸) designed for the study of condensing gases, adapted from an original design by Lee *et al.* (2016 ▸). During gas condensation, the cryofurnace was maintained at 95 K and connected to a gas delivery line, with the entire assembly placed within a cryofurnace. The tem­per­a­ture was controlled with the variable-tem­per­a­ture insert (VTI) of the cryofurnace – coupled to the sample environment with helium exchange gas, and a heater in a copper block above the sample can (tem­per­a­ture calibration information available in the supporting information). All data were collected without rotation of the cryostat. After sample condensation, the tem­per­a­ture was reduced to 5 K and a diffraction pattern was acquired for 2 h. Next, the tem­per­a­ture was increased to 195 K, and patterns were collected for 10 min at 5 K increments with the tem­per­a­ture stabilized at each point for 2 min before collection. At 100, 165 and 200 K, diffraction patterns were also acquired for 2 h.

Powder X-ray diffraction (PXRD) patterns were acquired on a Stoe Stadi-P diffractometer, operating in Debye–Scherrer geometry with an Mo *K*α_1_X-ray source (λ = 0.7107 Å) and three Mythen detectors in a stationary mode, covering a 2θ range of 0–55°. We used a 6 mm divergence slit (dimension parallel to the capillary sample) and a 3 mm × 0.5 mm collimator (dimensions parallel and perpendicular to the capillary, respectively). Using an Oxford Cryosystems nitrogen cryostream to control the tem­per­a­ture, the sample was condensed at 80 K from the gas phase into a thin-walled 0.5 mm quartz capillary (WJM-Glas) and subsequently flame-sealed to iso­late the sample from the atmosphere during PXRD measurements. The tem­per­a­ture was increased to 220 K at a rate of 2 K min^−1^, with diffraction measurements recorded simultaneously for 15 min in 2 K intervals. All data were acquired while rotating the capillary.

For structure solution, di­acet­y­lene was constructed as a rigid body using *Avogadro* software (Hanwell *et al.*, 2012 ▸) to minimize the number of degrees of freedom during the solution process. The rigid body was then implemented in the *Free Objects for Crystallography* program (*FOX* Version 2022.1) (Favre-Nicolin & Černý, 2002 ▸), where the Parallel Tempering algorithm was used to perform the structure solution using the PXRD pattern recorded at 132 K. The optimization resulted in a feasible structure that was then further refined *via* Rietveld refinement (Rietveld, 1969 ▸) with *GSAS-II* software by implementing di­acet­y­lene as a vector rigid body.

The parameters varied for the refinement against the PXRD pattern were scale factor, background function, including a single broad peak to account for the scattering of the borosilicate capillary, the lattice parameters, the orientation of the rigid body, origin position and isotropic displacement of the atoms (*U*_iso_). For the neutron powder diffraction pattern at 5 K, the same parameters were varied, as well as the crystallite size. Analysis of the two-dimensional plot from the neutron diffraction data collected at 5 K (see Fig. 1 in the supporting information) revealed some preferred orientation within the structure, which was then accounted for in the structure refinement.

### 2.2. Periodic-DFT calculation and micro-Raman spectroscopy

The resulting structure from the refinement against the laboratory X-ray data was used for geometry optimization and harmonic fre­quen­cy calculations applying density functional theory with periodic boundary (p-DFT). The *CRYSTAL17* software suite (Dovesi *et al.*, 2018 ▸; Pascale *et al.*, 2004 ▸) was used for these calculations at the PBE0 hybrid functional level of theory (Perdew *et al.*, 1996 ▸) supplemented with triple-ζ quality 6-311 G(d) basis sets, including D3(BJ) dispersion and dampening terms (Heyd *et al.*, 2005 ▸; Grimme, 2006 ▸). This level of theory has previously been shown to result in an accurate structure prediction and overall good agreement with ex­per­i­mental data of molecular crystal systems (Banks *et al.*, 2020 ▸). The coupled-perturbed Hartree–Fock/Kohn–Sham (CPHF/CPKS) approach was used to calculate the dielectric tensor and Raman intensities (Maschio *et al.*, 2013*a* ▸; Maschio *et al.*, 2013*b* ▸). The intensities of the polycrystalline Raman spectrum were calculated under the ex­per­i­mental conditions (Veithen *et al.*, 2005 ▸), including the laser wavelength and sample tem­per­a­ture (532 nm and 90 K, respectively). Total energy convergence for the geometry optimization and fre­quen­cy calculations were set to 10^−7^ and 10^−9^, respectively. The absence of imaginary frequencies confirmed convergence to a potential energy surface (PES) minimum. To account for the known overestimation of the calculated vibrational frequencies with the PBE0 functional (Pierre *et al.*, 2011 ▸), a scaling factor of 0.9518 was applied to correct for the harmonic approximation (Tikhonov *et al.*, 2024 ▸).

Experimental acquisition of the Raman spectra was done using a high-resolution confocal dispersive micro-Raman spectrometer (Horiba Jobin–Yvon LabRam HR). The spectrometer was equipped with a fre­quen­cy-doubled Nd:YAG laser (532 nm, 100 mW), an 1800 grooves/mm diffraction grating and a 50× Olympus BXFM objective. Spectra were acquired at a resolution of 0.4 cm^−1^ per detector pixel over the 50–4000 cm^−1^ fre­quen­cy range, with two spectral accumulations and 20 s acquisition per spectral region to improve the signal-to-noise ratio. The fre­quen­cy of the spectrometer was calibrated against a silicon standard reference peak at 520.7 cm^−1^.

A commercial Linkam LTS350 optical cryostage was mounted on the XYZ motorized translation stage underneath the objective, allowing for the sample tem­per­a­ture to be electronically controlled (to within ±0.1 K) by a resistive heating thermal block counterbalanced by a flow of liquid nitrogen. Before starting the ex­per­i­ment, the cryostage headspace was purged for 15 min with gaseous nitrogen at ∼330 K to remove residual atmospheric moisture. Then, di­acet­y­lene was deposited directly onto a microscope slide placed inside the cryostage at 200 K, to avoid concomitant crystallization of atmospheric carbon dioxide. The sample was then cooled to 90 K and the tem­per­a­ture increased in 10 K increments until 150 K, with an equilibration time of 2 min at each tem­per­a­ture point.

## 3. Results

### 3.1. Crystal structure determination

The neutron and X-ray powder diffraction (NPD and PXRD) patterns acquired at 5 and 132 K, respectively, were analysed using the *GSAS-II* software (Toby & Dreele, 2013 ▸), employing a combination of Gaussian and Lorentzian functions to fit the observed peaks. The autoindexing function of *GSAS-II* in both monoclinic and orthorhombic lattices was applied. The resulting M20 values and overall agreement between reflection positions and ex­per­i­mental peaks indicated a lattice in either the *P*222 or the *P*2 space group. Considering previous results on the di­acet­y­lene structure *via* polarized IR spectroscopy (Freund & Halford, 1965 ▸), which proposed di­acet­y­lene to have orthorhombic symmetry (however, no lattice parameters were proposed), indexing was progressed on that basis within *GSAS-II* and subsequent Pawley refinements (Pawley, 1981 ▸) were performed in the space group *P*222 (Fig. 2 in the supporting information).

It was noted, however, that small peaks are unaccounted for in the NPD and PXRD patterns. In the NPD pattern, the peaks at 39.5 and 41.5° (marked with an asterisk) are unaccounted for. For the PXRD pattern, a peak at 10.77 (marked with an asterisk) and other minor peaks at higher 2θ are also not accounted for in the Pawley fit. These impurity peaks likely suggest the presence of a known byproduct of the synthesis, identified as 2-chlorobut-1-en-3-yne (C_4_H_3_Cl) (Jolly *et al.*, 2014 ▸) by mass spectrometry with *m*/*z* 86 and 88, whose crystal structure is unknown. Differences in the peak positions are attributed to the intrinsic differences between the two techniques and the ex­per­i­mental conditions, *e.g.* the presence of preferred orientation. Therefore, the unaccounted peak was attributed to the presence of this known contaminant and was not considered further in the structure solution.

**Figure 1:**
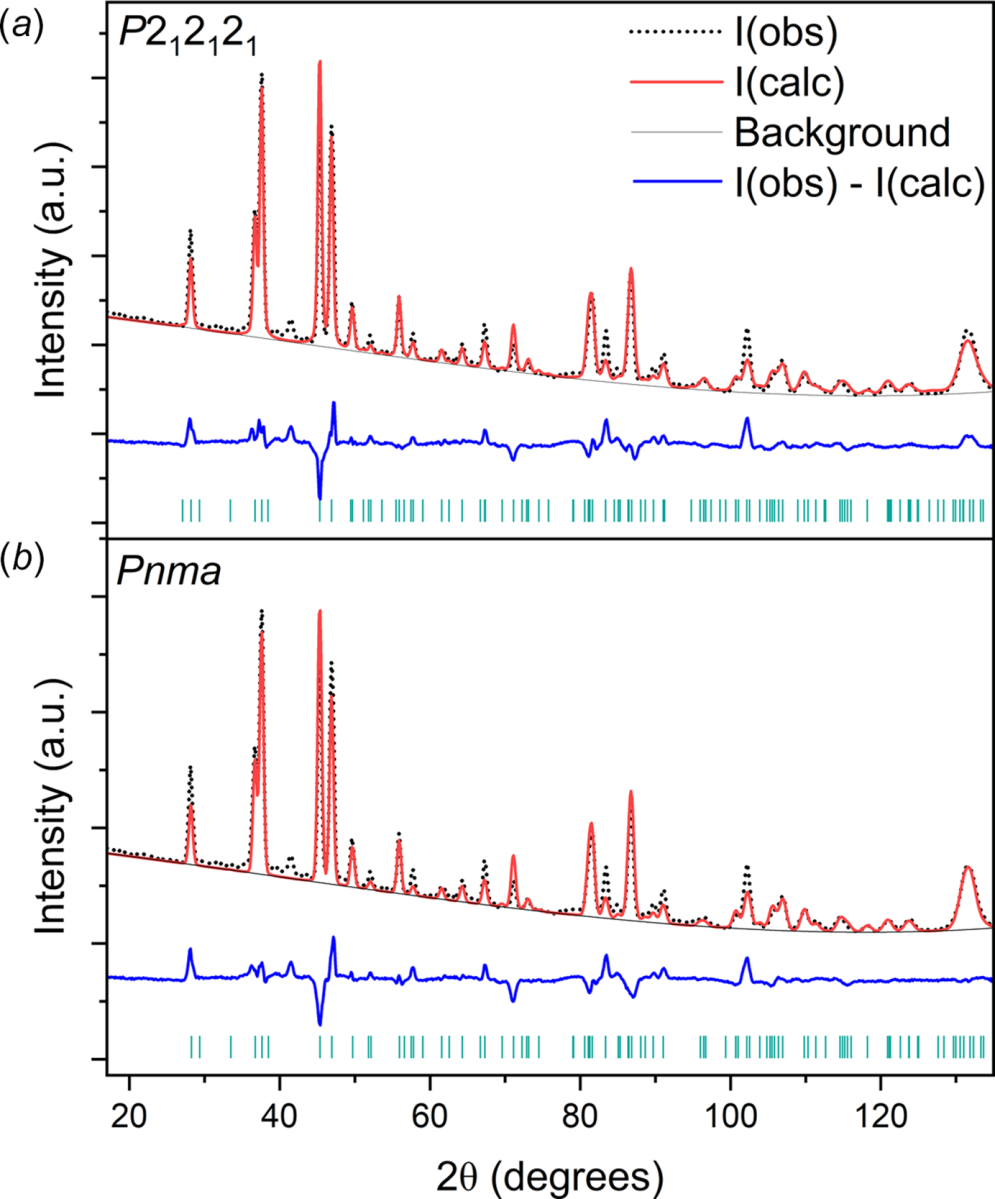
(*a*) Rietveld fit of the 5 K NPD pattern with the refined *P*2_1_2_1_2_1_ structure (*R*_wp_ = 3.49% and GoF = 4.41). (*b*) Rietveld fit of the 5 K NPD pattern with the refined *Pnma* structure (*R*_wp_ = 3.57% and GoF = 4.50). The blue line below the data indicates the difference between the observed (dotted black line) and calculated (red line; offset for clarity) patterns. The green tick marks indicate the position of expected reflections from the derived crystal structure.

Initial analysis of reflection conditions (*h*00, *h* = 2*n*; 0*k*0, *k* = 2*n*) and Le Bail refinements (Le Bail *et al.*, 1988 ▸) against the PXRD pattern at 132 K indicated *P*2_1_2_1_2_1_ as a possible space group (more information is provided in the supporting information). The volume of the unit cell obtained (329.51 Å^3^) and the space-group designation suggested the presence of four di­acet­y­lene molecules in the unit cell. Consequently, we first proceeded with structure solution in the space group *P*2_1_2_1_2_1_, where di­acet­y­lene was implemented as a rigid body with all its six atoms defined and placed in general positions. The molecule was placed with its centre of mass at the origin, and its position and orientation subsequently refined. The resulting Rietveld refinement against the PXRD pattern acquired at 132 K resulted in an *R*_wp_ value of 12.12% and a GoF of 2.42 (see Fig. 4 in the supporting information). Rietveld refinement was then performed against the neutron diffraction pattern collected at 5 K [Fig. 1 ▸(*a*)], resulting in *R*_wp_ = 3.49% and GoF = 4.41. The lattice parameters were determined to be *a* = 9.343 (2), *b* = 5.9859 (7), *c* = 5.6752 (13) Å and *V* = 317.39 (8) Å^3^, with a *U*_iso_ refined to 0.018 (2) Å^2^. The presence of preferred orientation was accounted for in the structure refinement with the [010] direction for a March–Dollase correction refined to 0.815 (7).

The molecular arrangement within the refined space group *P*2_1_2_1_2_1_ results in molecules adopting a slightly off-plane configuration, with *y* fractional coordinates close to 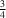; consequently, the possibility of describing the crystal structure in a higher-symmetry space group was investigated. The space group *P*2_1_2_1_2_1_ (No. 19) is a subgroup of *Pnma* (No. 62) where the inversion and mirror symmetries have been broken. Breaking the mirror symmetry can be achieved by moving the atoms from *y* = 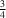 in *Pnma*, while retaining the same size unit cell. A structural remodel in *Pnma* was therefore created and tested against the neutron powder diffraction pattern acquired at 5 K [Fig. 1 ▸(*b*)]. This provided a satisfactory fit, and the final *R*_wp_ and GoF obtained were 3.57% and 4.50, respectively. The refined lattice parameters were: *a* = 9.348 (2), *b* = 5.9890 (6), *c* = 5.6746 (11) Å and *V* = 317.69 (6) Å^3^, with a *U*_iso_ value refined to 0.0219 (15) Å^2^ and a March–Dollase correction refined to 0.829 (7).

**Figure 2:**
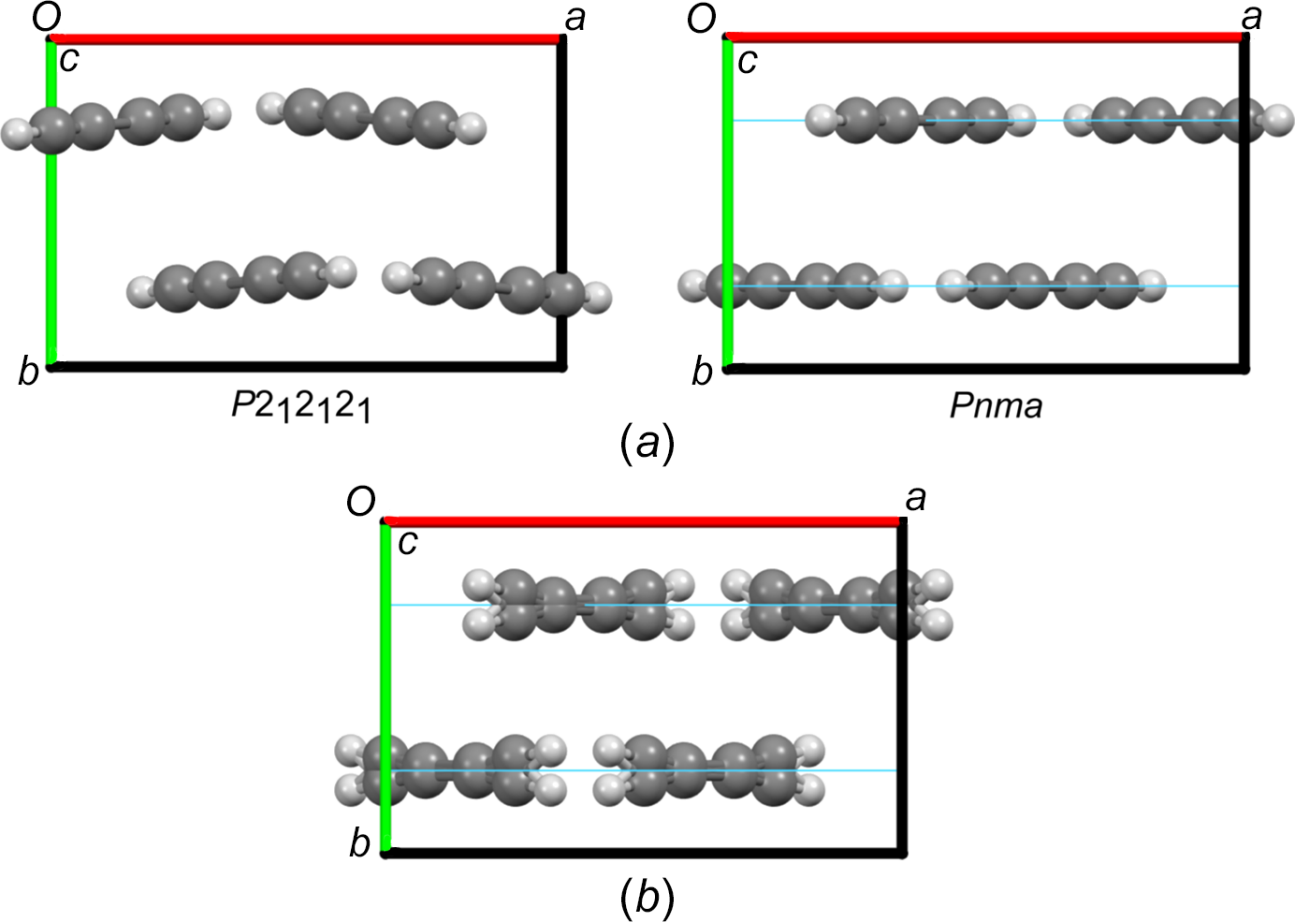
(*a*) Comparison between the refined structure at 5 K in the space group *P*2_1_2_1_2_1_ (left) and the molecular arrangement within the space group *Pnma* (right). The mirror planes in the *Pnma* structure are shown in light blue. (*b*) The molecular orientation after refining the molecular positions in the *Pnma* structure, resulting in a twofold disordered arrangement.

The molecular arrangement in the space group *P*2_1_2_1_2_1_ is shown in Fig. 2 ▸(*a*) (left). When the molecules are positioned precisely at *y* = 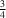 within the space group *Pnma*, they are placed on mirror planes [Fig. 2 ▸(*a*), right] without significantly altering the intermolecular interactions observed in the space group *P*2_1_2_1_2_1_.

Once the refinement of the molecular position is included in the model, the molecules shift off the mirror plane. This leads to a twofold positional disorder that improves the fit to the diffraction patterns [Table 1 ▸ and Fig. 2 ▸(*b*)]. Additional Rietveld refinements of the neutron diffraction patterns collected at 100, 165 and 200 K were also performed for both space groups under consideration, and the resulting *R*_wp_ and GoF values are shown in Table 1 ▸.

The presence of preferred orientation, impurities and the real-space resolution of the neutron diffractometer at λ = 2.41 Å imply that the resulting *R*_wp_ and GoF, although lower for the refinement in the space group *P*2_1_2_1_2_1_, might result from the higher positional degrees of freedom and overfitting of the data. Consequently, we concluded that the di­acet­y­lene crystal structure is better described by the space group *Pnma*. There remains the possibility that the structure is positionally disordered within the *Pnma* symmetry, but additional ex­per­i­ments would need to be conducted using, for example, inelastic neutron scattering.

**Figure 3:**
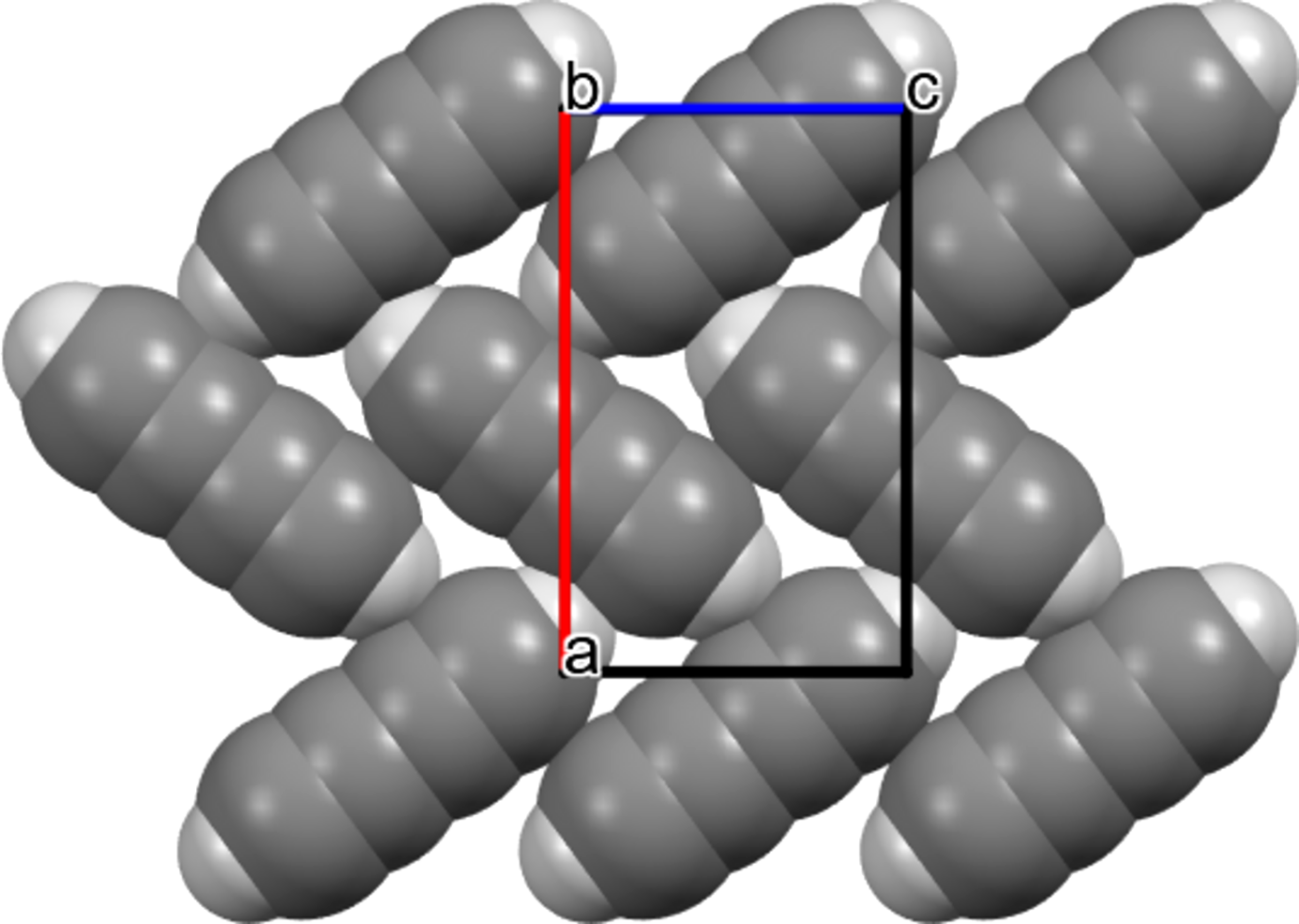
The molecular arrangement within the NPD refined structure at 5 K, viewed down the *b* axis, highlighting the main intermolecular C—H⋯C interaction.

The obtained crystal structure in the ordered space group *Pnma* shows that the di­acet­y­lene molecules form layers of almost perpendicular molecules (Fig. 3 ▸), resulting in an intricate network of intermolecular C—H⋯π interactions.

### 3.2. Thermal expansion and stability

**Figure 4:**
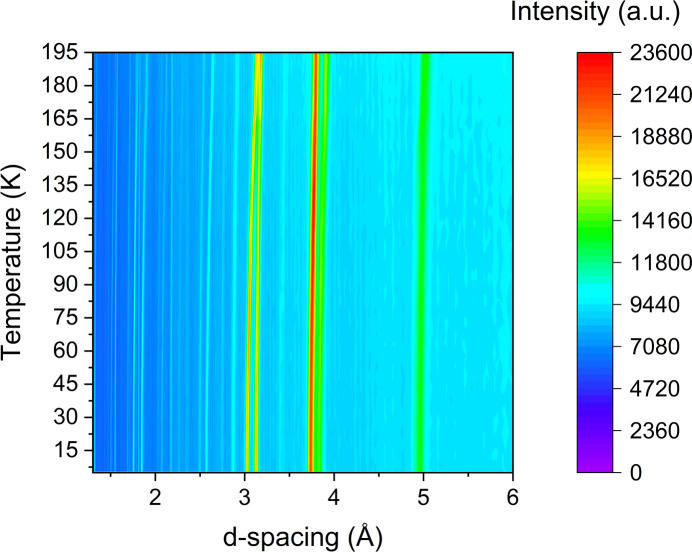
A thermodiffractogram of the neutron data from di­acet­y­lene ranging between 5 and 195 K. It can be seen from this plot that there are no phase transitions within this tem­per­a­ture range.

The thermodiffractogram of the neutron diffraction data taken from 5 to 195 K is shown in Fig. 4 ▸. A single phase was observed throughout the tem­per­a­ture range of this study, which extended to 220 K in the PXRD ex­per­i­ment (see Fig. 5 in the supporting information). Neutron diffraction data were not acquired above 200 K to preserve the ex­per­i­mental setup, due to the risk of polymerization in the liquid phase.

Using the determined crystal structure for di­acet­y­lene, Rietveld refinements were performed sequentially on the patterns collected by neutron diffraction from 5 to 195 K to observe changes in the unit-cell volumes and the dimensions of each crystallographic axis as a function of tem­per­a­ture. Detailed lattice parameters and unit-cell volumes derived from Rietveld refinements are listed in Table 1 of the supporting information.

**Figure 5:**
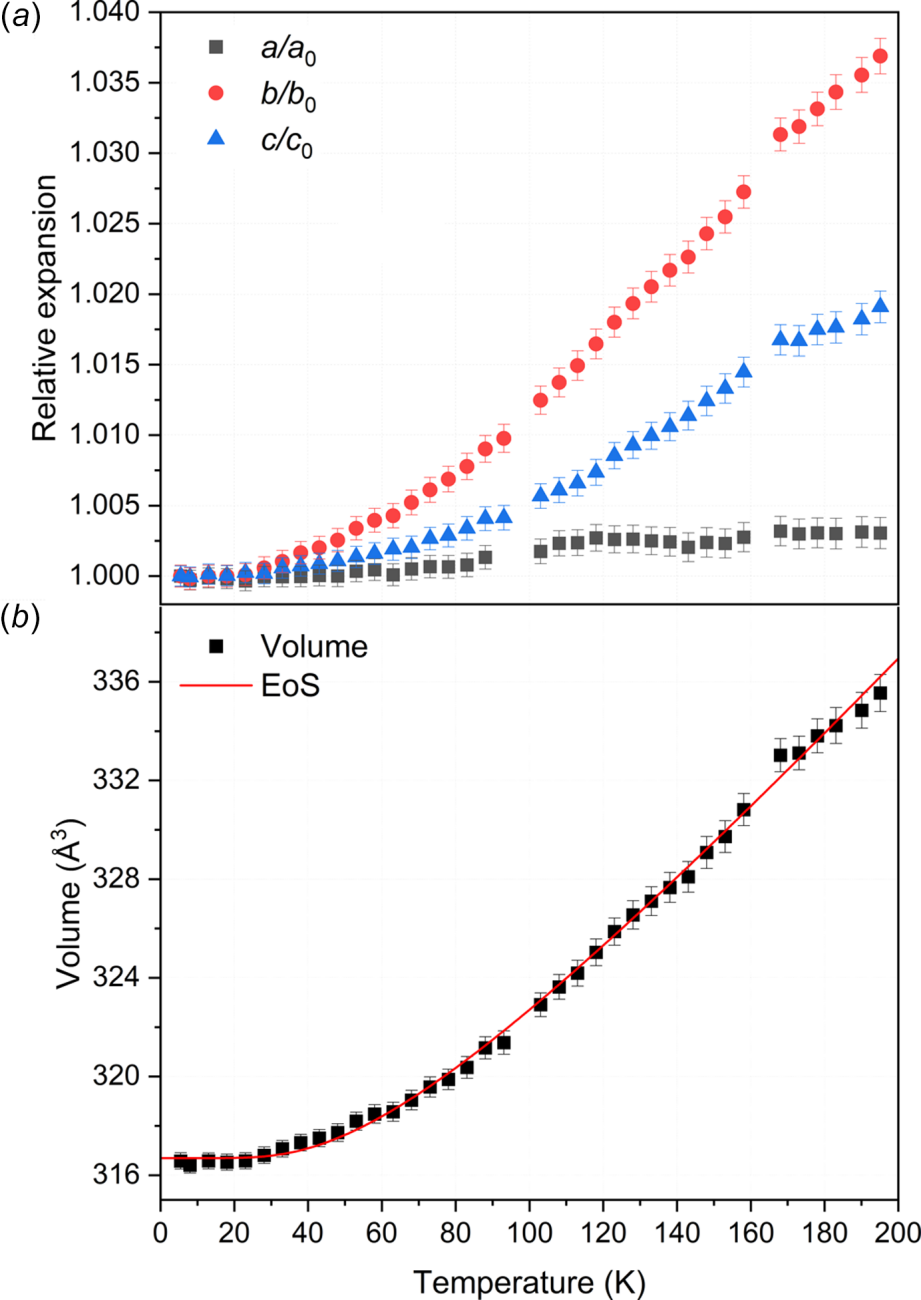
(*a*) Relative expansion of the *a*, *b* and *c* axes over the tem­per­a­ture range studied normalized to 5 K; a change in the thermal expansion rate is observed around 160 K. (*b*) Variation of the unit-cell volume with tem­per­a­ture, fitted with the Salje equation of state {*V*_0*T*_ = [*p*_0_ + *p*_1_θ_sat_coth(θ_sat_/*T*)]^3^} (Salje *et al.*, 1991 ▸).

The associated plot for the thermal expansion on each crystallographic axis and volume as a function of tem­per­a­ture is shown in Fig. 5 ▸. Here, an anisotropic behaviour is observed, reflecting the layered molecular arrangement parallel to the (010) plane, as shown in Fig. 2 ▸. In such an arrangement, there are no strong intermolecular interactions in the direction of the *b* axis, *i.e.* between the 0*k*0 planes. This reflects the higher relative expansion with increased tem­per­a­ture compared to the other two axes. The *a* and *c* axes contrarily, align with the molecular plane and their intermolecular interactions, resulting in a lower expansion with increased tem­per­a­ture. Fig. 6 in the supporting information shows how these interactions change with tem­per­a­ture, based on the Rietveld refinements against the NPD patterns acquired at 5, 100, 165 and 200 K.

The variations of the unit-cell volume with tem­per­a­ture were fitted to the Salje equation of states (Salje *et al.*, 1991 ▸) using the EoSFIT7 code (Angel *et al.*, 2014 ▸; Gonzalez-Platas *et al.*, 2016 ▸). The saturation tem­per­a­ture (θ_sat_) was refined to 104 (5) K, while the zero-tem­per­a­ture volume (*V*_0_) and *p*_1_ were refined to 317.25 (13) Å^3^ and 129 (4) × 10^5^ K^−1^, res­pec­tively.

### 3.3. Structure corroboration *via* vibrational spectroscopy and periodic DFT

**Figure 6:**
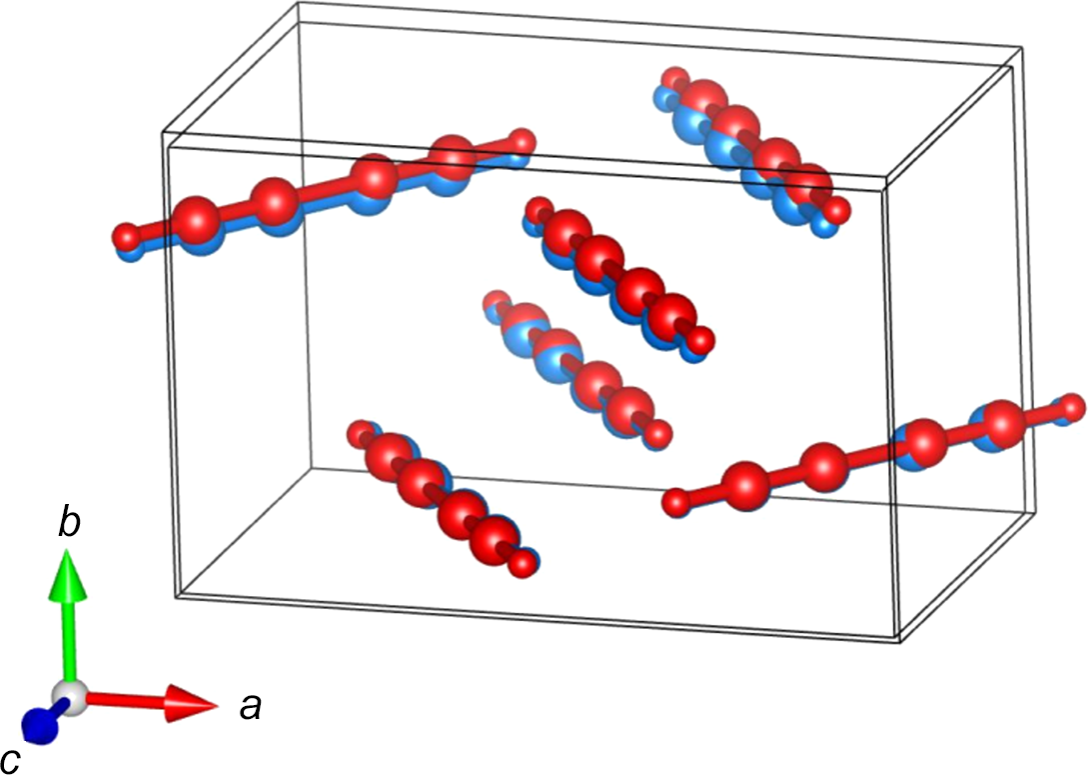
Comparison between the structure refined from the NPD data at 5 K (red; outer unit cell) with the output from geometry optimization (blue; inward unit cell).

Structural validation was sought from energy minimization calculations, which were then used to calculate the vibrational frequencies to compare with ex­per­i­mental vibrational spectroscopy results. Full geometry optimization of the obtained structure was done *via* p-DFT calculations, including dispersion corrections. The absence of imaginary frequencies during the fre­quen­cy calculation indicates that the final theoretical structure converged to a PES minimum, showing good agreement with the ex­per­i­mental structure (Fig. 6 ▸). A comparison between the two structures was conducted in the *Mercury* software (Macrae *et al.*, 2020 ▸) from the Cambridge Crystallographic Data Centre, using the *Crystal Packing Similarity* function. This resulted in an r.m.s. deviation of 0.175 for 12 molecules.

A comparison between the unit-cell parameters for the calculated and ex­per­i­mental structures is shown in Table 2 ▸. The optimized structure exhibited a deviation of less than 5% in the unit-cell parameters and approximately 10% in the volume compared with ex­per­i­mental values obtained at 5 K.

**Figure 7:**
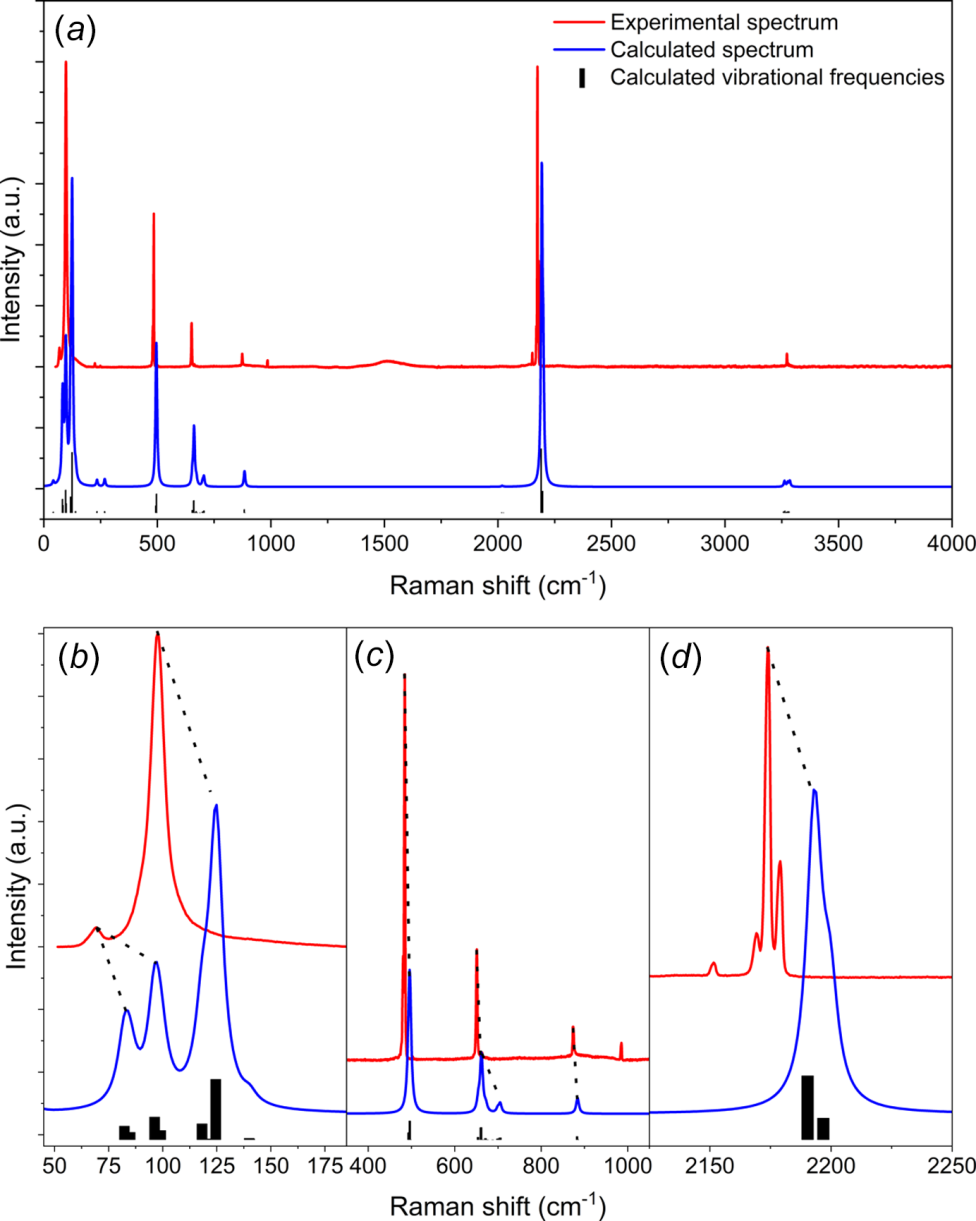
Comparison between the theoretical and ex­per­i­mental spectra of crystalline di­acet­y­lene at 90 K. (*a*) The top spectrum corresponds to that acquired ex­per­i­mentally (red), while the calculated Raman spectrum is displayed at the bottom (blue). The black bars correspond to the calculated vibrational modes. (*b*) The lower-fre­quen­cy range of the Raman spectra, where lattice vibrational modes are active. The correlation between corresponding bands is shown with black dotted lines. (*c*)/(*d*) Mid-fre­quen­cy range of the Raman spectra showing the fundamental vibrational modes.

To further validate the proposed structure, we performed p-DFT calculations to obtain simulated Raman and IR spectra (see the supporting information) to compare with ex­per­i­mental spectra. Comparison with ex­per­i­mental Raman spectra is especially important, as Raman spectroscopy provides information on the molecular arrangement and lattice vibrations within the crystalline structure, where the low-fre­quen­cy region can be used as a fingerprint for the crystal (Loudon, 2001 ▸). Here, the ex­per­i­mental Raman spectrum acquired at 90 K is compared with the p-DFT Raman spectrum simulated at the same tem­per­a­ture (Fig. 7 ▸). The observed and calculated Raman vibrational frequencies and mode assignments are provided in Table 3 ▸.

**Figure 8:**
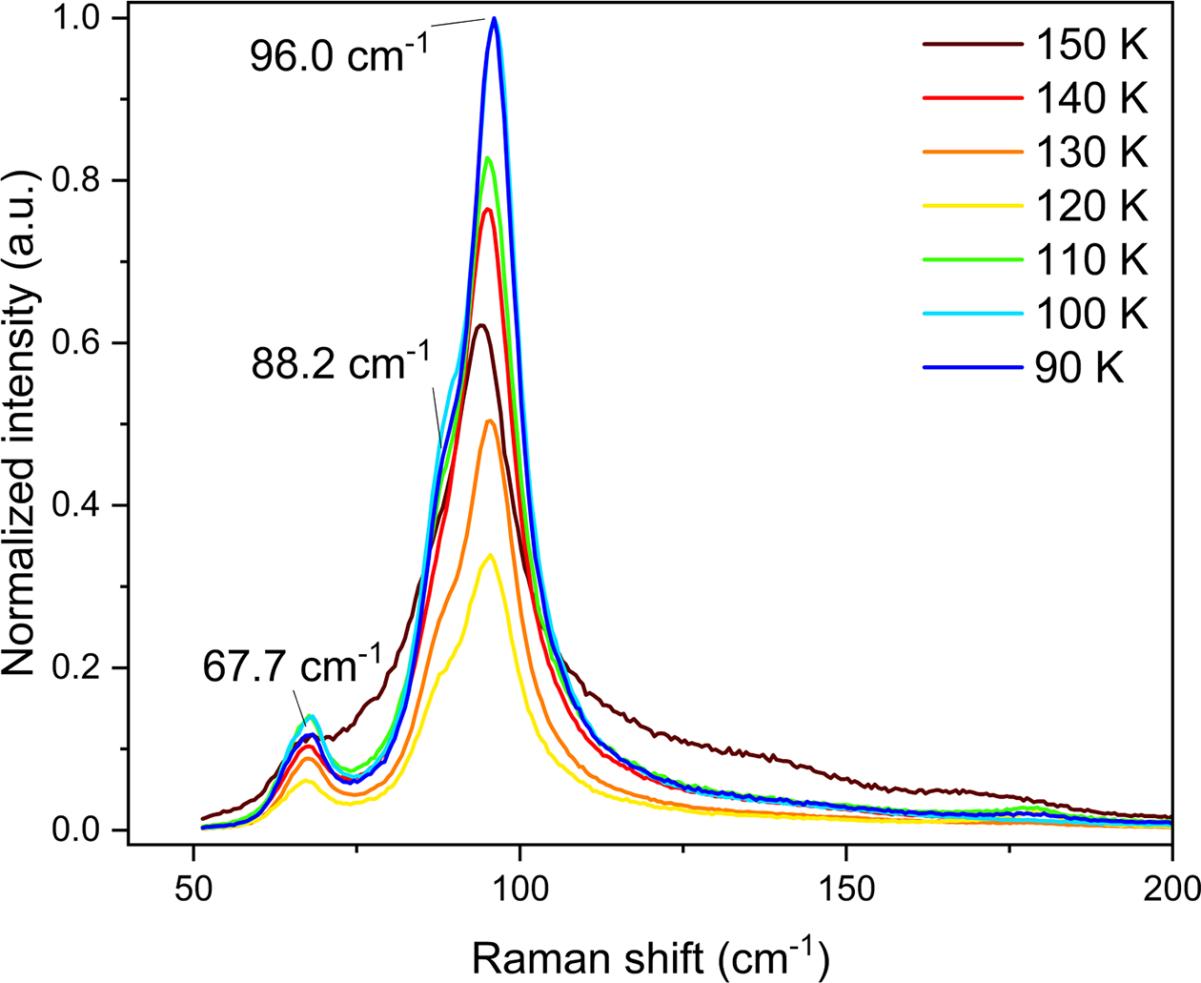
Expanded view of the lattice vibrational modes in the range 90–150 K. Two distinct bands can be identified at 96.0 and 67.7 cm^−1^, with a shoulder at 88.2 cm^−1^.

The calculated spectrum has an overall good agreement with the ex­per­i­mental spectrum, with a nearly one-to-one correspondence between the bands, in both their positions and intensities. All fundamental vibrational modes are accurately predicted, and anharmonic features (not calculated) explain the satellite features such as the band around 2170 cm^−1^, while the band at ∼1000 cm^−1^ can be attributed to an overtone of the band at 484 cm^−1^. The lattice vibrational modes calculated from the optimized crystal structure are also well predicted, with the bands around 98 and 82 cm^−1^ in the calculated spectrum likely corresponding to the broad band with low intensity at 69.9 cm^−1^ in the ex­per­i­mental spectrum. The 88.2 cm^−1^ shoulder to the band at 96 cm^−1^ also has a corresponding calculated band at 118 cm^−1^, more evident in Fig. 8 ▸.

In Table 3 ▸, our assigned vibrational modes and frequencies are also compared to those identified in previous ex­per­i­mental spectra of di­acet­y­lene in the liquid phase (Jones, 1952 ▸). The band shifts observed can be attributed to the different tem­per­a­tures and states (liquid and solid) in which the ex­per­i­ments were performed.

Fig. 8 ▸ shows the lattice vibrational region, where no significant changes occur between 90 and 150 K, with bands retaining their profile with minor shifts in position. Here, the varying intensities can be attributed to molecular motion within the lattice, without change in crystal phase. This is consistent with the single phase observed in the PXRD and neutron diffraction ex­per­i­ments.

## 4. Discussion

### 4.1. Comparison with the structure proposed from polarized IR spectroscopy

Using neutron and X-ray diffraction, this study demonstrates that di­acet­y­lene crystallizes in a single orthorhombic *Pnma* phase across the tem­per­a­ture range from 5 to 220 K. The molecular arrangement and anisotropic behaviour are consistent with previous spectroscopic findings.

The refined structure shows that di­acet­y­lene molecules form layers orthogonal to the *b* axis, while the molecular planes align along the *a* and *c* axes. These results agree with observations by Freund & Halford (1965 ▸) from polarized IR spectroscopy, where they identified absorptions of fundamental vibrational modes primarily occurring along two directions parallel to the molecular plane. Notably, no absorption was detected along the third direction, corresponding to the absence of dipole moment displacement in that orientation. This direction was later described as an axis orthogonal to the molecular plane. Furthermore, their results showed that both H atoms present in the molecule are involved in hydrogen interactions with the π-electron cloud of the C≡C bonds, supporting the molecular orientation in a coplanar arrangement shown in Fig. 3 ▸.

The planar configuration combined with the unit-cell volume refined here confirms a structure containing four molecules in the primitive unit cell, agreeing with the structure pro­posed by Freund & Halford (1965 ▸). In the space group *Pnma*, the molecules are placed on mirror planes, in contrast with results from Freund & Halford (1965 ▸) which suggested their placement in non-symmetric sites in the non-centrosymmetric orthorhombic space group *P*22_1_2_1_ (Freund & Halford, 1965 ▸). However, according to Kitaigorodskii’s rules, it is expected that the di­acet­y­lene crystal structure is centrosymmetric due to the molecular *D*_∞*h*_ point group, supporting our proposed structure in the space group *Pnma*.

### 4.2. Comparison with the crystal structure of acetylene

Acetylene is the simplest alkyne and exhibits two polymorphs at ambient pressure: an orthorhombic *Acam* crystal at tem­per­a­tures below 133 K, transitioning to a cubic *Pa*3 crystal above 133 K (Koski & Sándor, 1975 ▸; McMullan *et al.*, 1992 ▸). To compare with the refined crystal structure of di­acet­y­lene, the orthorhombic polymorph of acetylene was chosen due to the comparable space group and conditions under which both structures were analysed. As there are no low-tem­per­a­ture hydrogenous acetylene structures, the d-acetylene structure refined at 4.2 K [C_2_D_2_, *Acam* (Koski & Sándor, 1975 ▸>); CSD refcode ACETYL04] was compared with the structure of di­acet­y­lene refined at 5 K (*Pnma*, this work).

**Figure 9:**
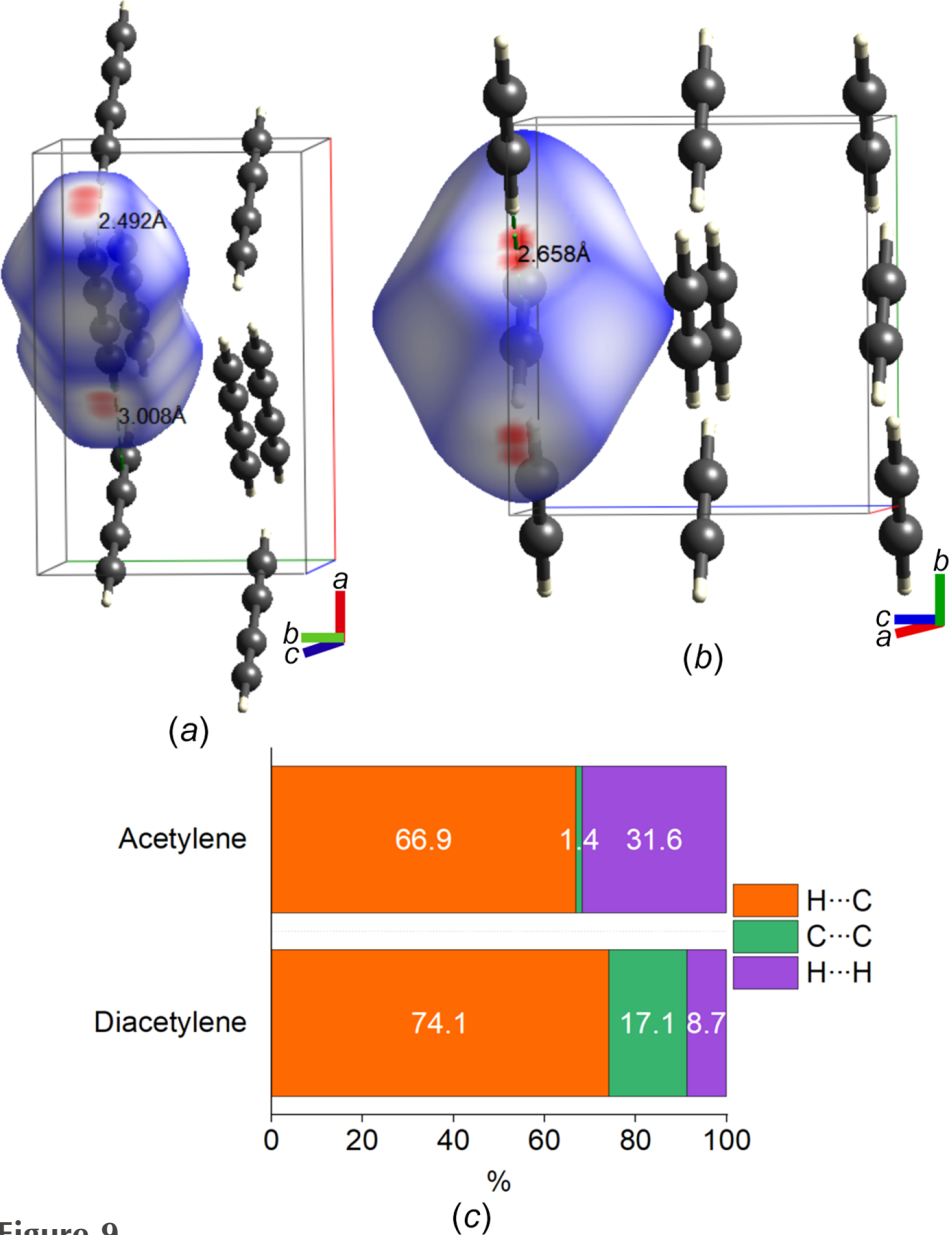
Hirshfeld surfaces of (*a*) di­acet­y­lene (refined at 5 K) and (*b*) acetylene [*Acam*; CSD refcode ACETYL04 (Koski & Sándor, 1975 ▸)] mapped with *d*_norm_ surfaces. (*c*) Relative contributions to the Hirshfeld surface area for the different close intermolecular contacts in acetylene and di­acet­y­lene.

To investigate intermolecular interactions and explore structural similarities, Hirshfeld surfaces (HSs) were con­structed using the *CrystalExplorer* program (Spackman *et al.*, 2021 ▸) (Fig. 9 ▸). These surfaces were mapped with the normalized contact distance (*d*_norm_) (Fig. 9 ▸), which considers the van der Waals (vdW) radii of the atoms and the distances to the nearest atoms, both internal and external to the surface (Spackman & Jayatilaka, 2009 ▸). Red regions on the HS indicate those contacts shorter than the sum of the vdW radii. White and blue regions represent contacts near and exceeding the length of the vdW limit, respectively (McKinnon *et al.*, 2007 ▸).

As shown in Figs. 9 ▸(*a*) and 9(*b*), both structures exhibit layered molecular arrangements with a slightly off-diagonal alignment. The closest intermolecular contacts highlighted in red arise from C—H⋯π interactions. Acetylene molecules lie parallel to the (001) plane and adopt a T-shaped geometry (Koski & Sándor, 1975 ▸), whereas di­acet­y­lene molecules align parallel to the (010) plane. The primary intermolecular interactions in both structures involve C—H⋯π interactions within the layers, with distances of 2.492 and 3.008 Å for di­acet­y­lenes placed on mirror planes in the *Pnma* structure. In contrast, the acetylene molecules lie in the mirror plane of *Acam* at positions of 2/*m* symmetry, resulting in a symmetric C—H⋯π interaction of 2.658 Å.

Fig. 9 ▸(*c*) details the relative contributions from the intermolecular contacts within the layers, extracted from the fingerprint plots shown in Fig. 8 in the supporting information. The C—H⋯π interactions dominate both structures, accounting for 74.1% of the contacts of di­acet­y­lene and 66.9% of the contacts of acetylene. Notable differences arise from the contributions of C⋯C and H⋯H close contacts. Diacetyl­ene’s layered arrangement results in an approximate interlayer distance of 3.1 Å and a higher crystal density of 1.0466 g cm^−3^ at 5 K, leading to a more closed-packed structure and consequent greater pro­por­tion of C⋯C interactions (17.1%). In contrast, the orthorhombic structure of acetylene has a lower density of 0.902 g cm^−3^ at 4.2 K, with minimal C⋯C interactions (1.4%). The increased H⋯H interactions in the structure of acetylene (31.6 *versus* 8.7%) can be attributed to the use of d-acetylene, as the larger vdW radius of deuterium influences the *d*_norm_ calculations.

The intermolecular interactions within di­acet­y­lene, in particular the C—H⋯π interactions, directly impact the thermal expansion observed in Fig. 5 ▸. Notably, the expansion along the *c* axis closely follows the variation in the intermolecular C—H⋯C distances, as shown in Fig. 6 in the supporting information. In contrast, the expansion along the *a* axis may result from a combination of two factors: the change in C—H⋯C distances and variation of the angle between the H atom and the C atoms involved in the triple bonds, resulting in the strong anisotropic behaviour observed. Additional ex­per­i­ments and theoretical simulations are needed to elucidate the molecular dynamics of the crystal and provide a more comprehensive description of the underlying mechanism governing the thermal expansion along the *a* axis.

### 4.3. Implications for Titan *in situ* exploration

Future *in situ* exploration, such as the Dragonfly rotorcraft (Barnes *et al.*, 2021 ▸), will encounter a range of materials on Titan’s surface. In preparation, attempts are being made to create a database of their properties, compiling information such as phase change and organic ice density (Yu *et al.*, 2023 ▸). Understanding these properties is critical for determining the physical state in which these compounds are likely to be found, depending on the type of terrain they encounter once reaching the surface (*i.e.* dry surface, lakes and seas).

While the crystal structures of some Titan organics remain unstudied ex­per­i­mentally, theoretical models were developed to predict some of these properties. For example, Yu *et al.* (2023 ▸) proposed that di­acet­y­lene’s density (ρ) varies with tem­per­a­ture (*T*) according to equation (3): 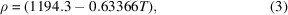
where ρ is measured in kg m^−3^ and *T* in Kelvin. Using this relationship, the predicted densities of di­acet­y­lene at 5, 100, 165 and 200 K are 1191.13, 1130.93, 1089.75 and 1067.57 kg m^−3^, respectively. In our study, however, the refined crystal structure yielded densities of 1046.6, 1029.2, 998.8 and 978.0 kg m^−3^ at 5, 100, 165 and 200 K, respectively, corresponding to deviations of 12.13, 8.99, 8.35 and 8.39% from the theoretical values.

To understand these discrepancies, we compared the di­acet­y­lene results with the published acetylene data [CSD refcode ACETYL06-10 (Koski & Sándor, 1975 ▸; McMullan *et al.*, 1992 ▸)], as Yu *et al.* (2023 ▸) similarly modelled the density of acetylene within the temperature range 77–188.15 K [equation (4)]: 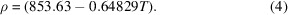


The resulting densities yielded an average difference of 10.07%, comparable to the deviations observed for di­acet­y­lene. For acetylene, the deviation likely arises from incorporating density data obtained *via* diffraction techniques and pycnometry in equation (4). Since pycnometry directly measures the volume of the condensed ice, it may include pore space (Yu *et al.*, 2023 ▸). The divergences observed for both hydrocarbons highlight the necessity for further ex­per­i­mental data on fundamental parameters that are essential for understanding physical and chemical processes occurring on planetary bodies such as Titan.

The structural similarities between di­acet­y­lene and acetylene may also imply a potential for di­acet­y­lene to participate in similar physicochemical processes. The layered arrangement and C—H⋯π interactions suggest that di­acet­y­lene could also cocrystallize with other hydrocarbons and nitriles. Previous studies have identified acetylene cocrystals with butane (Cable *et al.*, 2019 ▸), ammonia (Boese *et al.*, 2009 ▸; Cable *et al.*, 2018 ▸), acetonitrile (Kirchner *et al.*, 2010 ▸; Cable *et al.*, 2020 ▸), benzene (Boese *et al.*, 2003 ▸; Czaplinski *et al.*, 2020 ▸; Francis *et al.*, 2023 ▸), carbon dioxide (Gough & Rowat, 1998 ▸), pyridine (Czaplinski *et al.*, 2023 ▸) and propionitrile (Czaplinski *et al.*, 2025 ▸) under Titan-relevant conditions. Thus, these molecules are promising candidates for cocrystallization studies involving di­acet­y­lene.

Cocrystallization can also affect Titan’s surface chemistry by influencing the reactions that form complex organic molecules. For instance, previous studies indicate that the photochemistry of co-condensed pyridine and acetylene ices can form precursors to nitrogen-containing polycyclic aro­matic hydrocarbons. Contrarily, cocrystallization can hinder photodegradation and preserve the coformers (Lopes Cavalcante *et al.*, 2024 ▸). Both scenarios can potentially alter the range of materials found on Titan’s surface, highlighting the need for further ex­per­i­mental investigations, especially those involving di­acet­y­lene due to its absorption of longer wavelength UV photons (λ ≥ 300 nm) compared to acetylene (Couturier-Tamburelli *et al.*, 2015 ▸; Fleury *et al.*, 2019 ▸; Fleury *et al.*, 2024 ▸), where it could initiate photochemical reactions within the mixed ices and form more complex molecules.

## 5. Conclusion

This study presents the first crystallographic characterization and thermal expansion behaviour of di­acet­y­lene (1,3-butadiyne). Powder neutron and X-ray diffraction data indicate the formation of an orthorhombic structure (space group *Pnma*) with four molecules in the unit cell. The molecules are on mirror planes and arranged in coplanar layers within which C—H⋯π are the main intermolecular interactions. Variable-tem­per­a­ture ex­per­i­ments indicate that there are no phase transitions in the 5 to 220 K tem­per­a­ture range. Thermal expansion analysis reveals an anisotropic thermal behaviour, with larger expansion along the *b* direction, where no meaningful intermolecular interactions occur. Periodic-DFT calculations combined with Raman spectroscopy further support the proposed structure, which also agrees with previous spectroscopic considerations (Freund & Halford, 1965 ▸). Finally, comparison with the orthorhombic crystal structure of acetylene indicates structural and intermolecular interaction similarities. This implies that di­acet­y­lene may perform a similar role in forming cocrystals and complex organic molecules relevant to Titan’s surface chemistry.

## 6. Related literature

The following reference is cited in the supporting information: Fortes (2018) ▸.

## Supplementary Material

Crystal structure: contains datablock(s) NPD5K, restr_rigid_body_[], Rietveld+RB_NPD100K, restr_rigid_body_[]_1, Rietveld+RB_NPD165K, restr_rigid_body_[]_2, Rietveld+RB_NPD200K, restr_rigid_body_[]_3. DOI: 10.1107/S2052252525010486/fc5083sup1.cif

Supporting information. DOI: 10.1107/S2052252525010486/fc5083sup2.pdf

CCDC references: 2505575, 2513437, 2513438, 2513439

## Figures and Tables

**Table 1:** Resulting *R*_wp_ and GoF of the Rietveld refinement to the NPD patterns acquired at 5, 100, 165 and 200 K in the space groups *Pnma* and *P*2_1_2_1_2_1_

	*Pnma*	*Pnma* †	*P*2_1_2_1_2_1_
NPD pattern (K)	*R*_wp_ (%), GoF		
5	3.57, 4.50	3.48, 4.39	3.49, 4.41
100	3.71, 4.67	3.68, 4.65	3.55, 4.49
165	3.08, 3.89	2.88, 3.64	2.86, 3.61
200	2.89, 3.66	2.86, 3.62	2.88, 3.66

† Values after refining the molecular position within the structure.

**Table 2:** Comparison between the lattice parameters and unit-cell volumes of di­acet­y­lene at 5 K obtained from Rietveld refinements of the neutron diffraction data with the output from the geometry optimization

Structure	*a* (Å)	*b* (Å)	*c* (Å)	V (Å^3^)
NPD (5 K)	9.3478 (20)	5.9890 (6)	5.6746 (11)	317.69 (6)
p-DFT (0 K)	9.20585	5.72610	5.45640	287.626713

**Table 3:** Observed and calculated Raman vibrational frequencies (cm^−1^) and mode assignments for crystalline di­acet­y­lene

Assignment†	Mode	Exp. (90 K)	p-DFT (90 K)	Lit. (liquid ∼250 K)‡
ν_4_	CH asymmetric stretch	3281.4	3284.9	–
ν_1_	CH symmetric stretch	3273.1	3262.1	3293
ν_2_	CC triple-bond stretch	2173.9, 2178.9	2190.2, 2196.7	2172
ν_3_	CC single-bond stretch	873.5	883.4	877
ν_8_§	CCH bending	663.4	705.2	–
ν_6_	CCH bending	650.9	661.4	647
ν_7_	CCC bending	484.3	495.6	484
	Lattice vibration	96.0	124.5	
	Lattice vibration	88.2 (sh)	118.2	
	Lattice vibration	67.7	82.3, 98.2	

† Jones (1952 ▸) and Owen *et al.* (1987 ▸).

‡ Jones (1952 ▸).

§ Jolly *et al.* (2010 ▸).

## References

[bb1] Angel, R. J., Alvaro, M. & Gonzalez-Platas, J. (2014). *Z. Kristallogr. Cryst. Mater.* **229**, 405–419.

[bb2] Banks, P. A., Song, Z. & Ruggiero, M. T. (2020). *J. Infrared Milli. Terahz Waves*** 41**, 1411–1429.

[bb3] Barnes, J. W., Turtle, E. P., Trainer, M. G., Lorenz, R. D., MacKenzie, S. M., Brinckerhoff, W. B., Cable, M. L., Ernst, C. M., Freissinet, C., Hand, K. P., Hayes, A. G., Hörst, S. M., Johnson, J. R., Karkoschka, E., Lawrence, D. J., Le Gall, A., Lora, J. M., McKay, C. P., Miller, R. S., Murchie, S. L., Neish, C. D., Newman, C. E., Núñez, J., Panning, M. P., Parsons, A. M., Peplowski, P. N., Quick, L. C., Radebaugh, J., Rafkin, S. C. R., Shiraishi, H., Soderblom, J. M., Sotzen, K. S., Stickle, A. M., Stofan, E. R., Szopa, C., Tokano, T., Wagner, T., Wilson, C., Yingst, R. A., Zacny, K. & Stähler, S. C. (2021). *Planet. Sci. J.* **2**, 130.

[bb4] Barth, E. L. (2017). *Planet. Space Sci.* **137**, 20–31.

[bb5] Boese, R., Bläser, D. & Jansen, G. (2009). *J. Am. Chem. Soc.* **131**, 2104–2106. 10.1021/ja8059705 19199608

[bb6] Boese, R., Clark, T. & Gavezzotti, A. (2003). *Helv. Chim. Acta* **86**, 1085–1100.

[bb7] Brand, H. E. A., Gu, Q., Kimpton, J. A., Auchettl, R. & Ennis, C. (2020). *J. Synchrotron Rad.* **27**, 212–216. 10.1107/S1600577519015911 31868754

[bb8] Brochier, D. (1977). *Cryostat à température variable pour mesures neutroniques ou optiques.* Technical Report. Institut Laue–Langevin, Grenoble, France.

[bb9] Cable, M. L., Runčevski, T., Maynard-Casely, H. E., Vu, T. H. & Hodyss, R. (2021). *Acc. Chem. Res.* **54**, 3050–3059. 10.1021/acs.accounts.1c00250 34296607

[bb10] Cable, M. L., Vu, T. H., Malaska, M. J., Maynard-Casely, H. E., Choukroun, M. & Hodyss, R. (2019). *ACS Earth Space Chem.* **3**, 2808–2815.

[bb11] Cable, M. L., Vu, T. H., Malaska, M. J., Maynard-Casely, H. E., Choukroun, M. & Hodyss, R. (2020). *ACS Earth Space Chem.* **4**, 1375–1385.

[bb12] Cable, M. L., Vu, T. H., Maynard-Casely, H. E., Choukroun, M. & Hodyss, R. (2018). *ACS Earth Space Chem.* **2**, 366–375.

[bb13] Coustenis, A., Achterberg, R. K., Conrath, B. J., Jennings, D. E., Marten, A., Gautier, D., Nixon, C. A., Flasar, F. M., Teanby, N. A., Bézard, B., Samuelson, R. E., Carlson, R. C., Lellouch, E., Bjoraker, G. L., Romani, P. N., Taylor, F. W., Irwin, P. G. J., Fouchet, T., Hubert, A., Orton, G. S., Kunde, V. G., Vinatier, S., Mondellini, J., Abbas, M. M. & Courtin, R. (2007). *Icarus* **189**, 35–62.

[bb14] Couturier-Tamburelli, I., Piétri, N. & Gudipati, M. S. (2015). *A&A* **578**, A111.

[bb15] Czaplinski, E., Yu, X., Dzurilla, K. & Chevrier, V. (2020). *Planet. Sci. J.* **1**, 76.

[bb16] Czaplinski, E. C., Vu, T. H., Cable, M. L., Choukroun, M., Malaska, M. J. & Hodyss, R. (2023). *ACS Earth Space Chem.* **7**, 597–608. 10.1021/acsearthspacechem.2c00377 PMC10026175 36960425

[bb17] Czaplinski, E. C., Vu, T. H., Maynard-Casely, H., Ennis, C., Cable, M. L., Malaska, M. J. & Hodyss, R. (2025). *ACS Earth Space Chem.* In the press. 10.1021/acsearthspacechem.4c00262 PMC11849028 40008142

[bb56] De La Pierre, M., Orlando, R., Maschio, L., Doll, K., Ugliengo, P. & Dovesi, R. (2011). *J. Comput. Chem.* **32**, 1775–1784. 10.1002/jcc.21750 21469154

[bb18] Dovesi, R., Erba, A., Orlando, R., Zicovich–Wilson, C. M., Civalleri, B., Maschio, L., Rérat, M., Casassa, S., Baima, J., Salustro, S. & Kirtman, B. (2018). *WIREs Comput. Mol. Sci.* **8**, e1360.

[bb19] Favre-Nicolin, V. & Černý, R. (2002). *J. Appl. Cryst.* **35**, 734–743.

[bb20] Fleury, B., Gudipati, M. S. & Couturier-Tamburelli, I. (2024). *A&A* **684**, A1.

[bb21] Fleury, B., Gudipati, M. S., Couturier-Tamburelli, I. & Carrasco, N. (2019). *Icarus* **321**, 358–366.

[bb22] Fortes, A. D. (2018). *Acta Cryst.* B**74**, 196–216. 10.1107/S2052520618002159 29616994

[bb23] Francis, T. A., Maynard-Casely, H. E., Cable, M. L., Hodyss, R. & Ennis, C. (2023). *J. Phys. Chem. A* **127**, 2322–2335. 10.1021/acs.jpca.2c08791 36790472

[bb24] Freund, I. & Halford, R. S. (1965). *J. Chem. Phys.* **42**, 4131–4137.

[bb25] Gonzalez-Platas, J., Alvaro, M., Nestola, F. & Angel, R. (2016). *J. Appl. Cryst.* **49**, 1377–1382.

[bb26] Gough, T. E. & Rowat, T. E. (1998). *J. Chem. Phys.* **109**, 6809–6813.

[bb27] Grimme, S. (2006). *J. Comput. Chem.* **27**, 1787–1799. 10.1002/jcc.20495 16955487

[bb28] Hanwell, M. D., Curtis, D. E., Lonie, D. C., Vandermeersch, T., Zurek, E. & Hutchison, G. R. (2012). *J. Cheminform.* **4**, 1758–2946. 10.1186/1758-2946-4-17 PMC3542060 22889332

[bb29] Heyd, J., Peralta, J. E., Scuseria, G. E. & Martin, R. L. (2005). *J. Chem. Phys.* **123**, 174101. 10.1063/1.2085170 16375511

[bb30] Hörst, S. M. (2017). *J. Geophys. Res. Planets* **122**, 432–482.

[bb31] Huang, C., Zhang, F., Kaiser, R. I., Kislov, V. V., Mebel, A. M., Silva, R., Gichuhi, W. K. & Suits, A. G. (2010). *ApJ* **714**, 1249–1255.

[bb32] Jolly, A., Fayt, A., Benilan, Y., Jacquemart, D., Nixon, C. & Jennings, D. (2010). *ApJ* **714**, 852–859.

[bb33] Jolly, A., Manceron, L., Kwabia-Tchana, F., Benilan, Y. & Gazeau, M.-C. (2014). *Planet. Space Sci.* **97**, 60–64.

[bb34] Jones, A. V. (1952). *Proc. R. Soc. London Ser. A* **211**, 285–295.

[bb35] Khanna, R., Ospina, M. J. & Zhao, G. (1988). *Icarus* **73**, 527–535.

[bb36] Kirchner, M., Bläser, D. & Boese, R. (2010). *Chem. A Eur. J.* **16**, 2131–2146. 10.1002/chem.200901314 20029913

[bb37] Koski, H. K. & Sándor, E. (1975). *Acta Cryst.* B**31**, 350–353.

[bb38] Le Bail, A., Duroy, H. & Fourquet, J. (1988). *Mater. Res. Bull.* **23**, 447–452.

[bb39] Lee, S., Chevreau, H., Booth, N., Duyker, S. G., Ogilvie, S. H., Imperia, P. & Peterson, V. K. (2016). *J. Appl. Cryst.* **49**, 705–711.

[bb40] Lopes, R., Wall, S., Elachi, C., Birch, S. P., Corlies, P., Coustenis, A., Hayes, A., Hofgartner, J., Janssen, M. A., Kirk, R., LeGall, R., Lorenz, R. D., Lunine, J. I., Malaska, M. J., Mastroguiseppe, M., Mitri, G., Neish, C. D., Notarnicola, C., Paganelli, F., Paillou, P., Poggiali, V., Radebaugh, J., Rodriguez, S., Schoenfeld, A., Soderblom, J. M., Solomonidou, A., Stofan, E. R., Stiles, B. W., Tosi, F., Turtle, E. P., West, R. D., Wood, C. A., Zebker, H. A., Barnes, J. W., Casarano, D., Encrenaz, P., Farr, T., Grima, C., Hemingway, D., Karatekin, O., Lucas, A., Mitchell, K. L., Ori, G., Orosei, R., Ries, P., Riccio, D., Soderblom, L. A. & Zhang, Z. (2019). *Space Sci. Rev.* **215**, 1–50.

[bb41] Lopes Cavalcante, L., Czaplinski, E. C., Maynard-Casely, H. E., Cable, M. L., Chaouche-Mechidal, N., Hodyss, R. & Ennis, C. (2024). *Phys. Chem. Chem. Phys.* **26**, 26842–26856. 10.1039/d4cp03437f 39405048

[bb42] Loudon, R. (2001). *Adv. Phys.* **50**, 813–864.

[bb43] Macrae, C. F., Sovago, I., Cottrell, S. J., Galek, P. T. A., McCabe, P., Pidcock, E., Platings, M., Shields, G. P., Stevens, J. S., Towler, M. & Wood, P. A. (2020). *J. Appl. Cryst.* **53**, 226–235. 10.1107/S1600576719014092 PMC6998782 32047413

[bb44] Maschio, L., Kirtman, B., Rérat, M., Orlando, R. & Dovesi, R. (2013*a*). *J. Chem. Phys.* **139**, 164101. 10.1063/1.4824442 24181998

[bb45] Maschio, L., Kirtman, B., Rérat, M., Orlando, R. & Dovesi, R. (2013*b*). *J. Chem. Phys.* **139**, 164102. 10.1063/1.4824443 24181999

[bb46] Maynard-Casely, H. E., Cable, M. L., Malaska, M. J., Vu, T. H., Choukroun, M. & Hodyss, R. (2018). *Am. Mineral.* **103**, 343–349.

[bb47] Maynard-Casely, H. E., Hester, J. R. & Brand, H. E. A. (2020). *IUCrJ* **7**, 844–851. 10.1107/S2052252520007460 PMC7467175 32939276

[bb48] Maynard-Casely, H. E., Tobin, S. M., Wang, C.-W., Peterson, V. K., Hester, J. R. & Studer, A. J. (2025). *arXiv*, 2504.19429.

[bb49] McKinnon, J. J., Jayatilaka, D. & Spackman, M. A. (2007). *Chem. Commun.* pp. 3814–3816. 10.1039/b704980c 18217656

[bb50] McMullan, R. K., Kvick, Å. & Popelier, P. (1992). *Acta Cryst.* B**48**, 726–731.

[bb51] Nixon, C. A. (2024). *ACS Earth Space Chem.* **8**, 406–456. 10.1021/acsearthspacechem.2c00041 PMC10961852 38533193

[bb52] Owen, N. L., Smith, C. H. & Williams, G. A. (1987). *J. Mol. Struct.* **161**, 33–53.

[bb53] Pascale, F., Zicovich–Wilson, C. M., López Gejo, F., Civalleri, B., Orlando, R. & Dovesi, R. (2004). *J. Comput. Chem.* **25**, 888–897. 10.1002/jcc.20019 15011261

[bb54] Pawley, G. S. (1981). *J. Appl. Cryst.* **14**, 357–361.

[bb55] Perdew, J. P., Burke, K. & Ernzerhof, M. (1996). *Phys. Rev. Lett.* **77**, 3865–3868. 10.1103/PhysRevLett.77.3865 10062328

[bb57] Rietveld, H. M. (1969). *J. Appl. Cryst.* **2**, 65–71.

[bb58] Salje, E., Wruck, B. & Thomas, H. (1991). *Z. Phys. B Condens. Matter*** 82**, 399–404.

[bb59] Smith, N., Bénilan, Y. & Bruston, P. (1998). *Planet. Space Sci.* **46**, 1215–1220. 10.1016/0032-0633(94)00157-m 11538441

[bb60] Spackman, M. A. & Jayatilaka, D. (2009). *CrystEngComm* **11**, 19–32.

[bb61] Spackman, P. R., Turner, M. J., McKinnon, J. J., Wolff, S. K., Grimwood, D. J., Jayatilaka, D. & Spackman, M. A. (2021). *J. Appl. Cryst.* **54**, 1006–1011. 10.1107/S1600576721002910 PMC8202033 34188619

[bb62] Tikhonov, D. S., Gordiy, I., Iakovlev, D. A., Gorislav, A. A., Kalinin, M. A., Nikolenko, S. A., Malaskeevich, K. M., Yureva, K., Matsokin, N. A. & Schnell, M. (2024). *ChemPhysChem* **25**, e202400547. 10.1002/cphc.202400547 PMC11614367 39172051

[bb63] Toby, B. H. & Von Dreele, R. B. (2013). *J. Appl. Cryst.* **46**, 544–549.

[bb64] Veithen, M., Gonze, X. & Ghosez, P. (2005). *Phys. Rev. B* **71**, 125107.

[bb65] Vu, T. H., Maynard-Casely, H. E., Cable, M. L., Choukroun, M., Malaska, M. J. & Hodyss, R. (2022). *ACS Earth Space Chem.* **6**, 2274–2281.

[bb66] Yu, X., Yu, Y., Garver, J., Li, J., Hawthorn, A., Sciamma-O’Brien, E., Zhang, X. & Barth, E. (2023). *ApJS* **266**, 30.

[bb67] Yu, X., Yu, Y., Garver, J., Zhang, X. & McGuiggan, P. (2024). *Geophys. Res. Lett.* **51**, e2023G, L106156.

[bb68] Zhou, L., Kaiser, R. I. & Tokunaga, A. T. (2009). *Planet. Space Sci.* **57**, 830–835.

