# Supplementary material for: Crystal structure of di­acet­y­lene unveiled by X-ray and neutron diffraction, Raman spectroscopy and periodic DFT
Source: IUCrJ. 2026 Jan 1;13(Pt 1):94–104. doi: 10.1107/S2052252525010486 (PMC12809449; doi:10.1107/S2052252525010486)
Supplement: Supplementary file 2 [file m-13-00094-sup2.pdf]

IUCrJ

Volume 13 (2026)

Supporting information for article:

Crystal structure of diacetylene unveiled by X-ray and neutron diffraction, Raman spectroscopy and periodic DFT

Larissa Lopes Cavalcante, Helen E. Maynard-Casely, Morgan L. Cable, Samuel G. Duyker, Edith C. Fayolle, Robert Hodyss, Brendan J. Kennedy, Tuan H. Vu and Courtney Ennis

Supplementary Information: Crystal structure of diacetylene unveiled by X-ray and neutron diffraction, Raman spectroscopy, and periodic-DFT

Larissa Lopes Cavalcante,[a] Helen E. Maynard-Casely,[b]* Morgan L. Cable,[c] Samuel G. Duyker,[d] Edith C. Fayolle,[c] Robert Hodyss,[c] Brendan J. Kennedy,[e] Tuan H. Vu[c] and Courtney Ennis [a,f]*

[a]*Department of Chemistry, University of Otago, Dunedin 9054, New Zealand,* [b]*Australian Centre for Neutron Scattering, ANSTO, Kirrawee, NSW 2232, Australia,* [c]*Jet Propulsion Laboratory, California Institute of Technology, Pasadena, CA 91109, United States,* [d]*Sydney Analytical, Core Research Facilities, University of Sydney, NSW 2006, Australia,* [e]*School of Chemistry, University of Sydney, Sydney, NSW 2006, Australia, and* [f]*MacDiarmid Institute for Advanced Materials and Nanotechnology, Wellington 6140, New Zealand. E-mail: helenmc@ansto.gov.au, courtney.ennis@otago.ac.nz*

1. Calibration details - Sample environment for powder neutron diffraction experiments

Confirmation of measured temperatures and sample displacement was determined by delivering 2 ml D_2O to the sample position and undertaking a variable temperature experiment from 6 to 150 K in 2 K steps, the results of which were compared to previous D_2O studies (Fortes, 2018).

2. Additional powder diffraction data

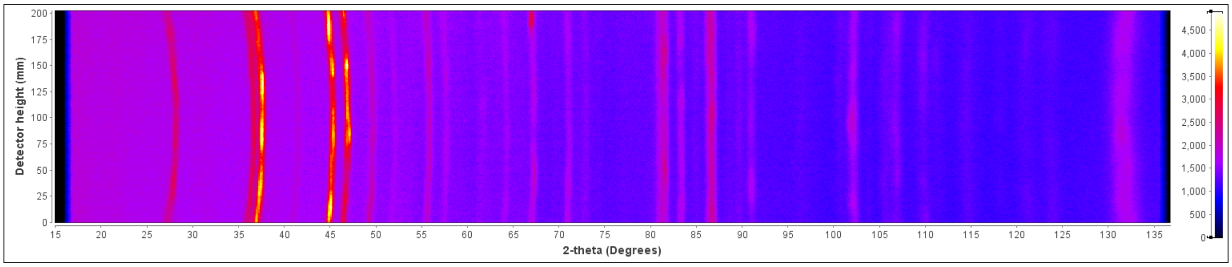

Fig. 1. Two-dimensional plot from the neutron diffraction data collected at 5 K. The heterogeneous intensity shown in some peaks indicates the presence of some preferred orientation, which was accounted for during structural refinement.

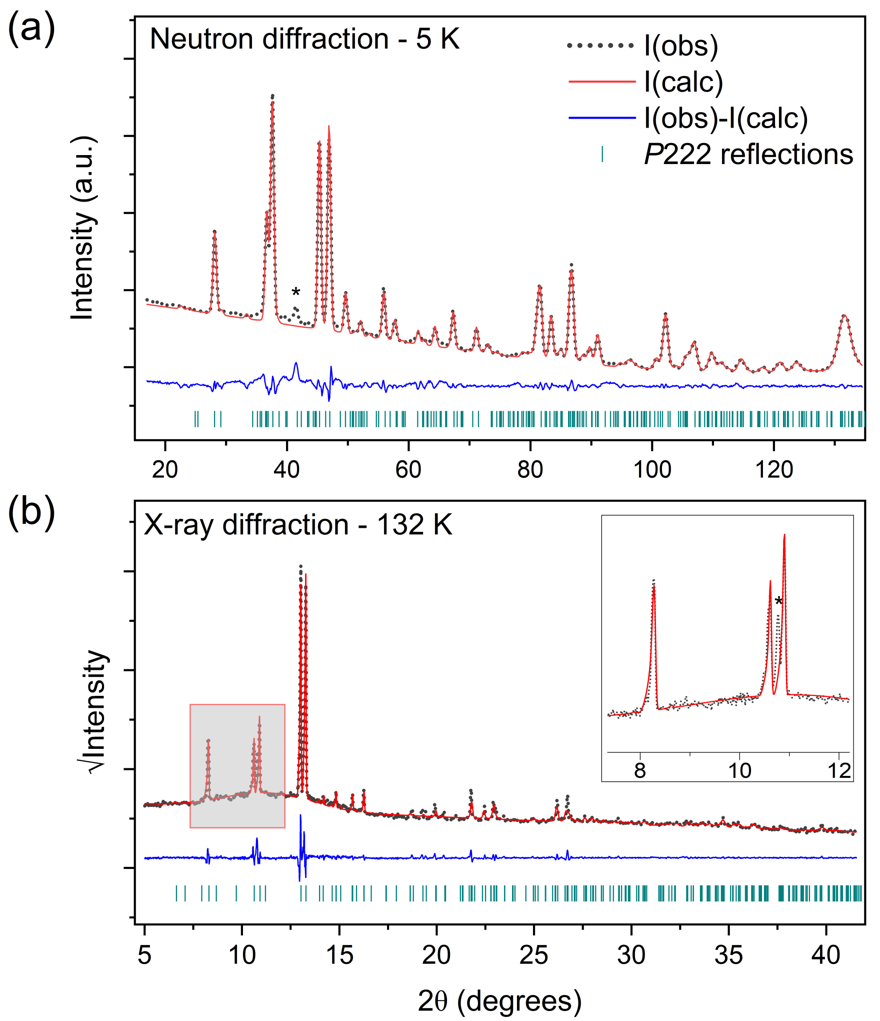

Fig. 2. (a) Pawley fit of the indexed 5 K pattern (grey dots) acquired by neutron diffraction. The refined orthorhombic $P222$ space group (red line) resulted in a = 9.356 Å, b = 5.991 Å, c = 5.682 Å, and a unit cell volume of 318.48 Å^3, with an R_{wp} of 1.66% and goodness of fit (GoF) of 2.19. (b) Pawley fit with the $P222$ (red line) for the pattern acquired by X-ray diffraction at 132 K (grey dots), yielding lattice parameters of a = 9.349 Å, b = 6.108 Å, c = 5.712 Å, and volume = 326.17 Å^3, with R_{wp} = 9.52% and GoF = 1.98. The blue line below the data indicates the difference between the observed (grey dots) and calculated patterns (red line). The green tick marks indicate the expected reflection position for the $P222$ space group. The peaks marked with a star likely correspond to contamination, as described in the text.

2.1. Initial structure refinement in $P2_12_12_1$ space group

Reflection conditions ($h00$, $h = 2n$; $0k0$, $k = 2$n) indicate two possible space groups: $P2_12_12$ or $P2_12_12_1$. The presence of the 003 and 005 reflections cannot be confirmed due to the low resolution of the neutron and X-ray diffraction patterns acquired, where their corresponding peaks in the diffraction patterns can also be attributed to the 112, and 031/311/204/302 reflections, respectively. The Le Bail refinements (Le Bail *et al.*, 1988) to the PXRD pattern at 132 K considering the two space groups are shown in Figure 3, which yielded a R_{wp} and GoF of: 13.382% and 2.73 for the fit with the $P2_12_12$ space group (Figure3(a)); 13.384% and 2.73 with the $P2_12_12_1$ space group (Figure3(b)).

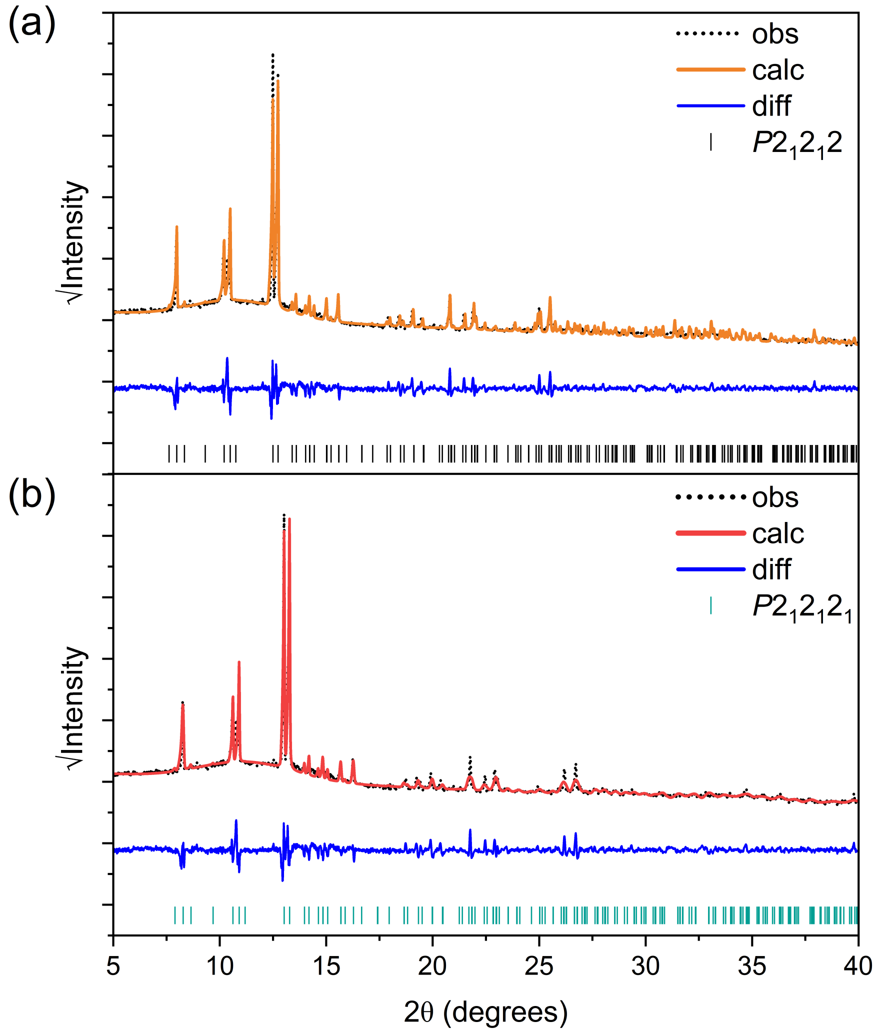

Fig. 3. Le Bail fits to the pattern acquired by X-ray diffraction at 132 K with the (a) $P2_12_12$ space group, resulting in the lattice parameters: $a = 9.359$ Å, $b = 6.122$ Å, $c = 5.742$ Å, and volume of 328.98 Å^3, $R_{wp} = 13.382\%$ and GoF = 2.73; (b) $P2_12_12_1$ space group, resulting in the lattice parameters: $a = 9.373$ Å, $b = 6.126$ Å, $c = 5.739$ Å, and volume of 329.59 Å^3, $R_{wp} = 13.384\%$ and GoF = 2.73. The blue line below the data indicates the difference between the observed (grey dots) and calculated patterns (blue and red lines). The tick marks indicate the expected reflection position for each space group.

Considering the similar results for the Le Bail fit, structural refinement proceeded with the space group $P2_12_12_1$. The resulting Rietveld refinement is shown in Figure 4, with final unit cell parameters of: $a = 9.3488(16)$ Å, $b = 6.1102(10)$ Å, $c = 5.7190(14)$ Å, and volume = 326.73(17) Å^3. The parameters varied for this refinement were scale

factor, background function including a single broad peak to account for the scattering of the borosilicate capillary, the lattice parameters, the orientation of the rigid body, origin position and isotropic displacement of the atoms (refined to U_{iso} of 0.199(7) Å^2). These resulted in an R_{wp} of 12.121% and a GoF of 2.42.

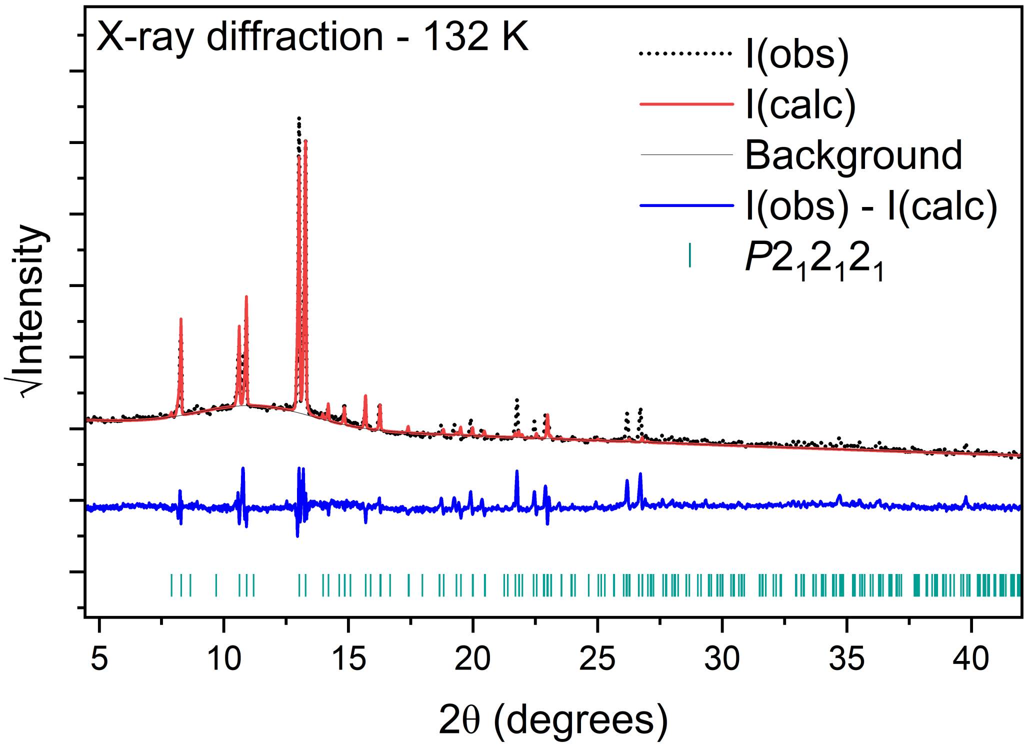

Fig. 4. X-ray powder diffraction pattern of diacetylene at 132 K (dotted grey line) and Rietveld refinement (red) (R_{wp} = 12.121%; GoF = 2.42). The blue line below the data indicates the difference between the observed (dotted black line) and calculated patterns (red line; offset for clarity). The green tick marks indicate the position of expected reflections from the derived crystal structure (space group $P2_12_12_1$).

The presence of preferred orientation was accounted for in the structure refinement with the [010] direction for a March-Dollase correction refined to 0.817(4).

2.2. Thermal expansion and stability

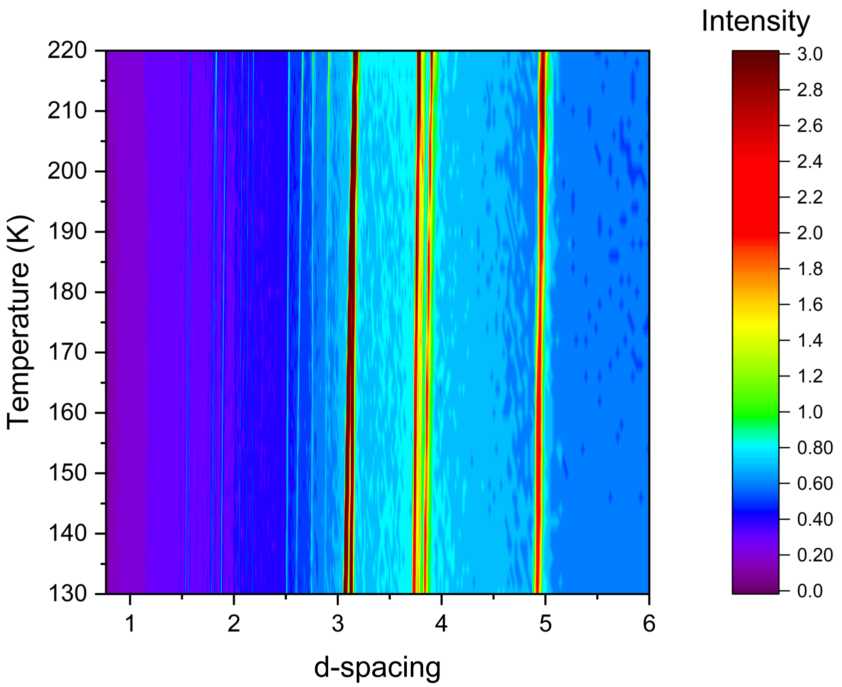

Fig. 5. A thermodiffractogram of the X-ray data from diacetylene ranging between 130 and 220 K. It can be seen from this plot that there are no phase transitions within this temperature range, in agreement with the results from the neutron diffraction experiment.

Table 1. *Lattice parameters and unit cell volumes of diacetylene over the temperatures 5 to 195 K at 1 bar obtained from Rietveld refinements against the neutron diffraction data*

Temperature (K)	a (Å)	b (Å)	c (Å)	V (Å^3)
5.5	9.340 (5)	5.984 (3)	5.672 (2)	317.0 (4)
8.0	9.337 (5)	5.982 (3)	5.671 (2)	316.8 (4)
13.0	9.339 (5)	5.984 (3)	5.672 (2)	317.0 (4)
18.0	9.338 (5)	5.983 (3)	5.672 (2)	316.9 (4)
23.0	9.337 (5)	5.984 (3)	5.673 (2)	317.0 (4)
28.0	9.340 (5)	5.987 (3)	5.673 (2)	317.2 (4)
33.0	9.340 (5)	5.990 (3)	5.675 (2)	317.5 (4)
38.0	9.340 (5)	5.993 (3)	5.676 (2)	317.7 (4)
43.0	9.340 (5)	5.995 (3)	5.677 (3)	317.9 (4)
48.0	9.340 (6)	5.998 (3)	5.678 (3)	318.1 (4)
53.0	9.344 (6)	6.003 (3)	5.679 (3)	318.5 (4)
58.0	9.344 (6)	6.007 (3)	5.681 (3)	318.8 (4)
63.0	9.341 (6)	6.008 (4)	5.682 (3)	318.9 (5)
68.1	9.345 (6)	6.013 (4)	5.683 (3)	319.4 (5)
73.1	9.347 (6)	6.018 (4)	5.686 (3)	319.9 (5)
78.1	9.347 (6)	6.022 (4)	5.687 (3)	320.1 (5)
83.1	9.348 (6)	6.027 (4)	5.690 (3)	320.6 (5)
88.1	9.354 (7)	6.034 (4)	5.694 (3)	321.4 (5)
93.1	9.353 (7)	6.037 (4)	5.693 (3)	321.4 (5)
98.0	9.360 (6)	6.052 (4)	5.700 (3)	322.9 (5)
103.0	9.357 (6)	6.051 (4)	5.701 (3)	322.8 (5)
108.1	9.363 (7)	6.057 (4)	5.703 (3)	323.4 (6)
113.1	9.364 (7)	6.063 (4)	5.705 (3)	323.9 (6)
118.1	9.367 (7)	6.072 (5)	5.709 (3)	324.7 (6)
123.1	9.367 (7)	6.084 (5)	5.718 (3)	325.9 (6)
128.1	9.368 (7)	6.091 (5)	5.722 (3)	326.5 (6)
133.1	9.368 (7)	6.098 (4)	5.725 (3)	327.0 (6)
138.1	9.368 (7)	6.107 (5)	5.729 (4)	327.8 (6)
143.1	9.365 (7)	6.114 (5)	5.734 (4)	328.3 (6)
148.1	9.368 (7)	6.123 (5)	5.740 (4)	329.2 (6)
153.1	9.367 (7)	6.133 (5)	5.746 (4)	330.1 (6)
158.1	9.372 (7)	6.144 (5)	5.753 (4)	331.2 (7)
163.0	9.377 (7)	6.168 (5)	5.766 (4)	333.5 (6)
168.1	9.375 (7)	6.172 (5)	5.767 (4)	333.7 (7)
173.1	9.373 (7)	6.175 (5)	5.766 (4)	333.7 (6)
178.1	9.376 (7)	6.186 (5)	5.773 (4)	334.9 (6)
183.1	9.377 (7)	6.195 (5)	5.773 (4)	335.4 (7)
190.1	9.380 (7)	6.205 (5)	5.779 (4)	336.3 (7)
195.1	9.378 (7)	6.213 (5)	5.783 (4)	337.0 (7)

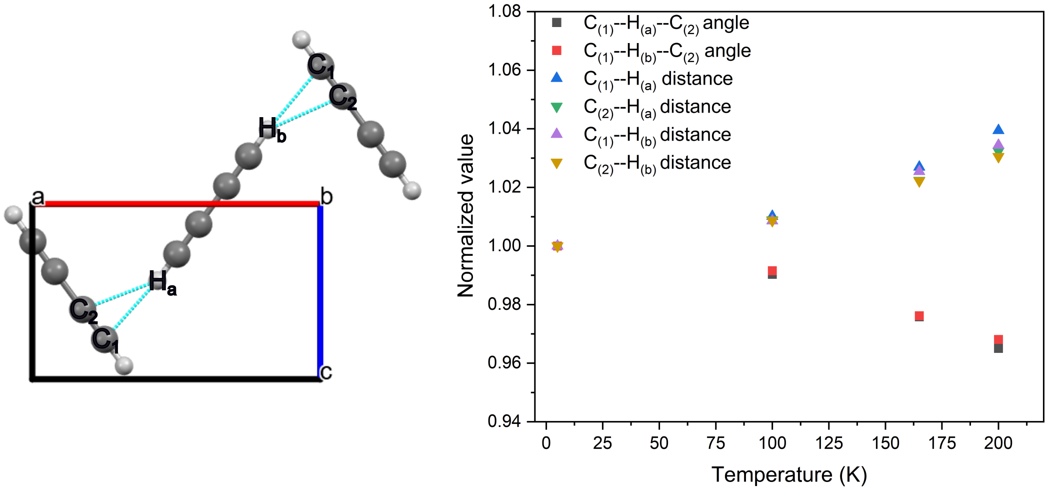

Fig. 6. Intermolecular interactions in the (010) plane (left). Relative change in intermolecular distances (triangles) and angles (square) between the hydrogens and the carbons from the C≡C bonds with increased temperature from 5 to 200 K from the NPD patterns acquired for 2 h at 5, 100, 165 and 200 K (right).

3. Additional Spectroscopy data: Experimental *vs* pDFT calculated infrared spectra

3.1. Gas deposition: formation of molecular thin films

The thin films of molecular ices were produced in a stainless-steel high vacuum chamber evacuated to a base pressure of 10^{-9} torr using turbomolecular pumps backed by dry diaphragm pumps. A KBr optical window attached to the second stage of a closed-cycle helium cryocooler (Sumitomo RDK-101D) was cooled to 18 K to act as an infrared transparent substrate. The surface temperature was monitored by a silicon diode positioned directly above the substrate linked to a temperature control unit (Lakeshore 330) that reports to an accuracy of ±0.5 K. A 50 Ω heater positioned beneath the substrate allowed temperature control to 300 K. Deposition of the molecular thin films was performed by introducing desired quantities of synthesized diacetylene through a gas manifold connected to a leak valve before the chamber. Inline and independent mass flow controllers (MKC Type 1179A) allowed finely tuned gas depo-

sition over 15-30 minutes at 18 K. A vacuum-compatible rotary platform allowed the optical window to be rotated 90° for transmission IR experiments and UV photolysis experiments. For IR data acquisition, mid-IR spectra (600-4000 cm^{-1}) were collected by a Thermo iG50 FTIR spectrometer equipped with an internal SiC thermal light source and a liquid N_2 cooled mercury-cadmium-telluride detector. The spectra were recorded in a sequential loop using OMNIC 9 software, with each spectrum averaged over 64 individual scans at 4 cm^{-1} resolution or 1 cm^{-1}.

3.2. Comparison between experimental and calculated spectra

Figure 7 shows the infrared spectrum acquired at 90 K (green) compared with the p-DFT calculated spectrum (red). The experimental bands are well described by the calculated spectrum both in position and intensity. The figure inserts highlight the profile of some bands for the CH stretch and bending modes. For the stretch region, the calculated band is broad and corresponds to overlapping vibrational frequencies (shown as black bars), being equivalent to the two experimental bands in that region (Table 2). In the case of the bending modes, the calculated spectrum shows two broad bands that correspond to the three overlapping bands seen in the experimental spectrum. A full description of the band positions, their assignment, mode and comparison with literature results are shown in Table 2. An experimental band at 2337 cm^{-1} likely corresponds to carbon dioxide contamination.

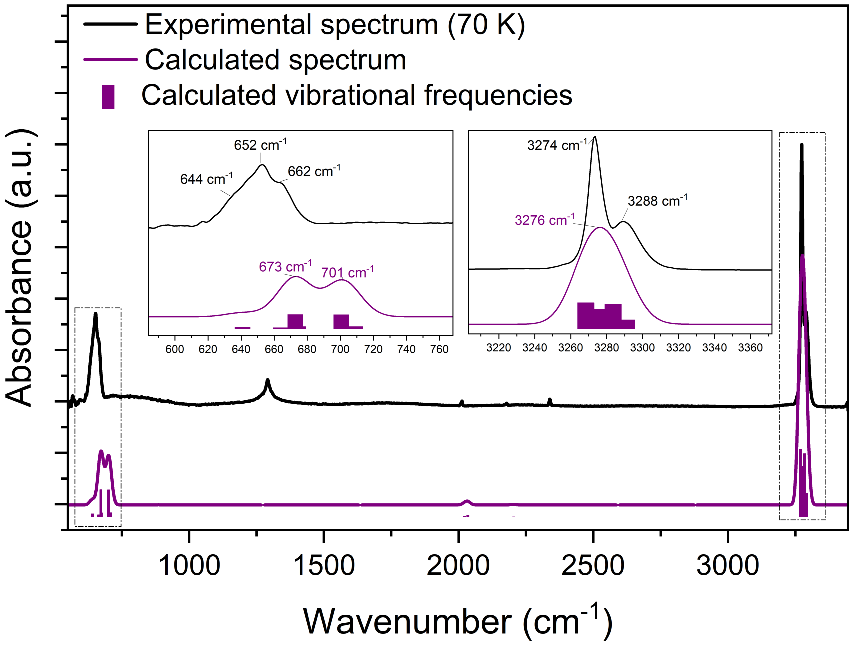

Fig. 7. Comparison between the theoretical (red) and experimental infrared spectra of crystalline diacetylene at 90 K (green). The black bars correspond to the calculated vibrational modes.

Table 2. *Observed and calculated Infrared vibrational frequencies* (cm^{-1}) *and Mode assignments for crystalline diacetylene*

Assignment	Mode	Exp. (90 K)	p-DFT	Lit. (94 K)†
ν_4	CH stretch	3288, 3274	3276	3278, 3273
ν_5	C≡C stretch	2012	2032	2013, 2007
ν_{6+8}	Combination	1289		1292
ν_8	CH bend	662, 652	701, 673	674
ν_6	CH bend	644		627‡

† (Zhou *et al.*, 2009)
‡ (Khanna *et al.*, 1988)

4. Hirshfeld surface fingerprint plots

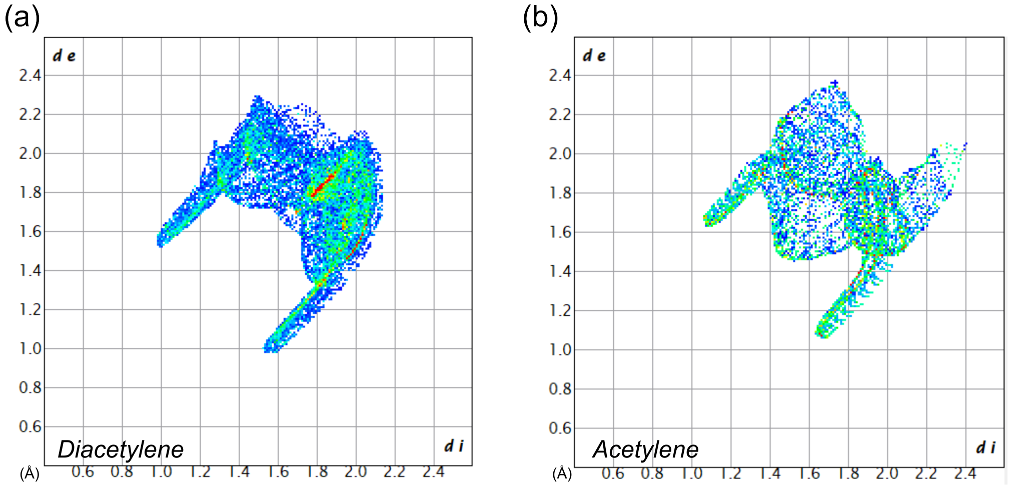

Fig. 8. Full fingerprint plots for (a) diacetylene (refined at 5 K) and (b) acetylene (*Acam*; CSD refcode: ACETYL04 (Koski & Sándor, 1975)).

References

Fortes, A. D. (2018). *Acta Crystallographica Section B: Structural Science, Crystal Engineering and Materials*, **74**(2), 196–216.

Khanna, R., Ospina, M. J. & Zhao, G. (1988). *Icarus*, **73**(3), 527–535.

Koski, H. K. & Sándor, E. (1975). *Acta Crystallographica Section B*, **31**(2), 350–353.

Le Bail, A., Duroy, H. & Fourquet, J. (1988). *Materials Research Bulletin*, **23**(3), 447–452. https://www.sciencedirect.com/science/article/pii/0025540888900190

Zhou, L., Kaiser, R. I. & Tokunaga, A. T. (2009). *Planetary and Space Science*, **57**(7), 830–835.
